# Underwater SLAM Meets Deep Learning: Challenges, Multi-Sensor Integration, and Future Directions

**DOI:** 10.3390/s25113258

**Published:** 2025-05-22

**Authors:** Mohamed Heshmat, Lyes Saad Saoud, Muayad Abujabal, Atif Sultan, Mahmoud Elmezain, Lakmal Seneviratne, Irfan Hussain

**Affiliations:** Khalifa University Center for Autonomous and Robotic Systems, Khalifa University, Abu Dhabi P.O. Box 127788, United Arab Emirateslyes.saoud@ku.ac.ae (L.S.S.);

**Keywords:** underwater robotics, simultaneous localization and mapping (SLAM), deep learning (DL), underwater SLAM, DL-based SLAM, underwater image enhancement

## Abstract

The underwater domain presents unique challenges and opportunities for scientific exploration, resource extraction, and environmental monitoring. Autonomous underwater vehicles (AUVs) rely on simultaneous localization and mapping (SLAM) for real-time navigation and mapping in these complex environments. However, traditional SLAM techniques face significant obstacles, including poor visibility, dynamic lighting conditions, sensor noise, and water-induced distortions, all of which degrade the accuracy and robustness of underwater navigation systems. Recent advances in deep learning (DL) have introduced powerful solutions to overcome these challenges. DL techniques enhance underwater SLAM by improving feature extraction, image denoising, distortion correction, and sensor fusion. This survey provides a comprehensive analysis of the latest developments in DL-enhanced SLAM for underwater applications, categorizing approaches based on their methodologies, sensor dependencies, and integration with deep learning models. We critically evaluate the benefits and limitations of existing techniques, highlighting key innovations and unresolved challenges. In addition, we introduce a novel classification framework for underwater SLAM based on its integration with underwater wireless sensor networks (UWSNs). UWSNs offer a collaborative framework that enhances localization, mapping, and real-time data sharing among AUVs by leveraging acoustic communication and distributed sensing. Our proposed taxonomy provides new insights into how communication-aware SLAM methodologies can improve navigation accuracy and operational efficiency in underwater environments. Furthermore, we discuss emerging research trends, including the use of transformer-based architectures, multi-modal sensor fusion, lightweight neural networks for real-time deployment, and self-supervised learning techniques. By identifying gaps in current research and outlining potential directions for future work, this survey serves as a valuable reference for researchers and engineers striving to develop robust and adaptive underwater SLAM solutions. Our findings aim to inspire further advancements in autonomous underwater exploration, supporting critical applications in marine science, deep-sea resource management, and environmental conservation.

## 1. Introduction

The vast and largely unexplored marine environment plays a pivotal role in scientific exploration, resource management, and environmental conservation. Autonomous underwater vehicles (AUVs) are essential for a wide range of applications, including coral reef monitoring, offshore infrastructure inspection, deep-sea exploration, and underwater archaeology. A fundamental requirement for these robotic systems is robust and precise navigation, which is largely enabled by simultaneous localization and mapping (SLAM). SLAM enables AUVs to construct spatial maps while simultaneously estimating their position within the environment [1]. This capability is crucial for executing complex missions with minimal human intervention, particularly in hazardous or remote underwater settings.

Despite the success of SLAM in terrestrial environments, its adaptation to underwater domains presents significant challenges. These include low visibility due to light absorption and scattering, sensor noise, dynamic lighting conditions, and distortions caused by the water column [2]. Unlike in air, where visual and LiDAR-based SLAM systems are widely used, underwater SLAM relies on a combination of sonar, acoustic signals, inertial sensors, and vision-based approaches, each with inherent limitations. These constraints degrade the accuracy of feature extraction, loop closure detection, and trajectory estimation, which are critical components of SLAM pipelines.

To mitigate these challenges, deep learning (DL) has emerged as a transformative tool in underwater SLAM. Convolutional neural networks (CNNs) and transformer-based architectures have demonstrated remarkable improvements in feature extraction, denoising, and data-driven sensor fusion, addressing many limitations of traditional methods [3]. DL-powered models facilitate robust perception in low-visibility conditions, enhance map consistency, and improve loop closure detection, significantly augmenting SLAM performance in underwater environments [4]. Additionally, multi-modal approaches integrating vision, sonar, and inertial data have proven effective in compensating for sensor deficiencies, enabling more resilient localization and mapping systems.

In addition to enhancing SLAM, an emerging research direction is the integration of underwater wireless sensor networks (UWSNs). UWSNs provide a distributed sensing framework where multiple AUVs and sensor nodes collaboratively share localization and mapping data via acoustic communication [5]. By incorporating UWSN-based SLAM, navigation accuracy and robustness can be improved, especially in large-scale, multi-agent underwater operations.

This survey presents a comprehensive review of DL-driven underwater SLAM, addressing key challenges, recent innovations, and future research directions. We propose a novel classification framework that integrates UWSN-based SLAM methodologies, highlighting their impact on collaborative navigation and large-scale mapping. By synthesizing the latest advancements in deep learning, sensor fusion, and communication-aware SLAM, this work aims to bridge the gap between theoretical research and practical applications, paving the way for next-generation autonomous underwater navigation systems.

### 1.1. Motivations

The study of underwater SLAM is not only a technical challenge but also a necessity for a range of real-world applications with significant scientific, economic, and environmental implications. Autonomous underwater exploration is crucial for tasks such as oceanographic mapping, deep-sea mining, disaster response, conducting inspections of underwater structures, and marine ecosystem conservation. However, reliable SLAM in underwater environments remains a formidable challenge due to fundamental constraints such as visibility degradation, sensor drift, and high localization uncertainty.

**Figure 1:**
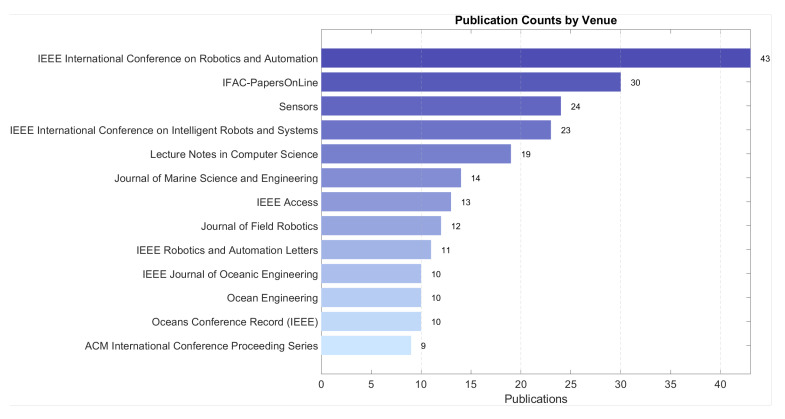
Number of publications in underwater SLAM research by publication venue.

**Figure 2:**
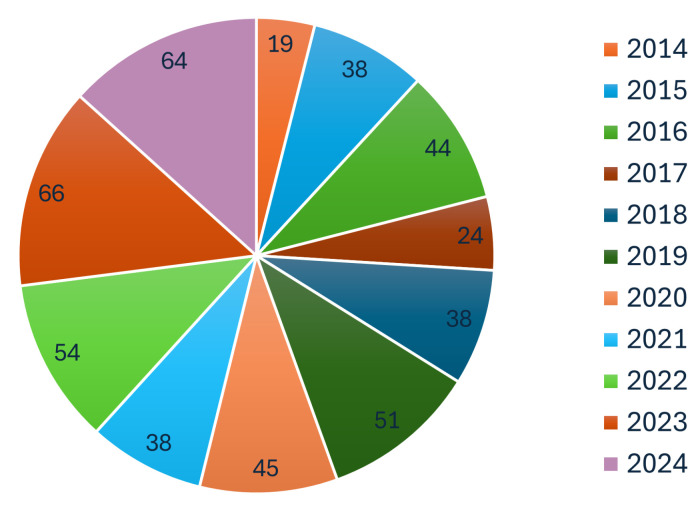
Number of publications per year in underwater SLAM research over the last decade.

Figure 1 shows that underwater SLAM research has been extensively published in leading robotics conferences and journals, underscoring its central importance. The growing interest in underwater SLAM is further reflected in the steady increase in SLAM-related publications over the past decade, as illustrated in Figure 2. This trend highlights the rapid advancements in underwater SLAM technologies and their expanding applications.

Traditional SLAM methodologies, which rely on visual and geometric feature extraction, struggle to maintain accuracy in underwater conditions where textures are often repetitive, feature points are scarce, and sensor noise is prevalent. Moreover, acoustic-based localization, while promising, suffers from latency and bandwidth limitations. These issues necessitate a paradigm shift towards deep learning-enhanced SLAM, where neural networks can learn domain-specific features, improve sensor fusion, and enable robust mapping under extreme conditions.

Beyond the technical challenges, underwater SLAM requires optimization for real-world deployment, where power efficiency, computational constraints, and adaptability to dynamic environments are critical factors. Many AUVs operate on limited battery resources, and real-time SLAM inference demands lightweight, energy-efficient DL models. Additionally, collaborative SLAM systems leveraging UWSNs offer new opportunities for large-scale, multi-robot navigation, but they introduce challenges related to communication latency and synchronization. Given these considerations, this survey is motivated by three key factors:

- **The need for improved SLAM accuracy in extreme underwater conditions:** Enhancing robustness against visibility constraints, dynamic environments, and sensor noise is essential for real-world deployments.
- **The growing role of deep learning in enhancing underwater SLAM pipelines:** DL-based techniques provide solutions for feature extraction, sensor fusion, and loop closure detection, outperforming traditional methods in challenging underwater conditions.
- **The integration of UWSNs for scalable, cooperative SLAM:** Multi-agent localization and distributed mapping offer promising improvements but require addressing communication constraints and synchronization challenges.

### 1.2. Contributions

This paper provides a detailed exploration of the evolution of SLAM methodologies, with a particular emphasis on the integration of deep learning techniques across different stages of the SLAM pipeline. Our primary contributions are as follows:

- **Comprehensive review of DL-driven underwater SLAM methodologies:** We systematically analyze state-of-the-art approaches that leverage CNNs, transformers, and multi-modal sensor fusion techniques to enhance SLAM accuracy and robustness in underwater settings.
- **Identification and analysis of key challenges:** We investigate the major technical barriers in underwater SLAM, including lack of visibility, sensor drift, and acoustic communication constraints, providing insights into how DL methods address these issues.
- **Novel classification of SLAM approaches integrating UWSNs:** We introduce a new taxonomy for underwater SLAM based on its integration with UWSNs, emphasizing the role of distributed sensing and collaborative navigation in improving mapping accuracy and operational efficiency.
- **Critical evaluation and future research directions:** We outline open challenges and promising avenues for future research, including self-supervised learning for feature extraction, real-time deployment of lightweight DL models, and large-scale SLAM solutions for extended-duration underwater missions.

By providing a structured synthesis of recent advancements, this survey serves as a key resource for researchers and practitioners in underwater robotics, deep learning, and autonomous navigation. Beyond addressing technical challenges, it highlights the broader significance of underwater SLAM, emphasizing its role in environmental monitoring, resource exploration, and multi-robot coordination. By bridging research gaps and showcasing the transformative impact of deep learning and networked SLAM techniques, this review paves the way for the next generation of autonomous underwater systems. Through an in-depth evaluation of state-of-the-art methodologies, we offer critical insights into the future of underwater robotics, driving innovation toward more reliable, efficient, and adaptive navigation in complex marine environments.

The remainder of this paper is structured as follows. Section 2 provides an overview of the *core principles of SLAM*, introducing the SLAM front-end (feature-based and direct methods) and the SLAM back-end (filtering-based and optimization-based approaches). Section 3, *Underwater SLAM: Background and Overview*, discusses key challenges, tracing the evolution of underwater SLAM, and emphasizing the importance of UWSNs. Section 4 presents our *proposed SLAM classification based on UWSNs*, highlighting traditional classifications and introducing our new framework that integrates networking considerations. Section 5 focuses on the *sensors driving underwater SLAM*, including vision, acoustic, laser-based, and multi-modal sensing systems. Section 6 addresses the *practical challenges in underwater SLAM*, covering communication constraints, computational resources, and energy efficiency. Section 7 reviews *recent advances in deep learning-based SLAM*, examining feature extraction, pose estimation, loop closure detection, mapping, and 3D reconstruction, as well as integration with UWSNs and computational efficiency. Section 8 offers an *underwater SLAM systems evaluation and comparison*, discussing relevant metrics and comparing traditional versus DL-based methods. Section 9 identifies *research gaps and opportunities*, including the need for new datasets, the potential for deep reinforcement learning, improved 3D reconstruction and semantic mapping, large language model integration, dynamic environment handling, communication optimization in cooperative SLAM, new sensor technologies, and practical deployment challenges. Finally, Section 10, *Conclusions*, summarizes the key findings, reiterates the significance of the proposed UWSN-based classification, and provides a forward-looking perspective on underwater SLAM research.

## 2. Core Principles of SLAM: Front-End and Back-End

**Figure 3:**
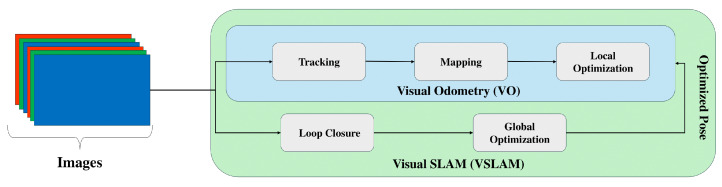
Overview of visual odometry and VSLAM systems. VSLAM is an extension of visual odometry, incorporating loop closure detection and global optimization to refine the overall trajectory and minimize accumulated drift. (Adopted from Gadipudi et al. [7]).

SLAM is a fundamental problem in robotics, concerned with building a map of an unknown environment while simultaneously determining the robot’s location within that map [6]. The core challenge lies in overcoming sensor limitations and noise while incrementally building a consistent map of the environment. SLAM systems aim to improve both localization and mapping by incorporating them together. In contrast, visual odometry (VO) focuses mainly on estimating the robot’s motion by analyzing camera images without creating a map. The critical difference is SLAM’s ability to build and optimize a map, providing a broader spatial understanding. VO is limited to tracking movement relative to the environment without mapping it. Figure 3 illustrates the difference between the SLAM and VO algorithms.

SLAM systems are typically divided into two main modules: front-end and back-end. The front-end processes raw sensor data to extract meaningful features or observations, which involves filtering sensor noise, detecting landmarks, extracting features, or interpreting depth information. The front-end’s primary goal is to convert raw data into a format that localization and mapping modules can use. On the other hand, the back-end takes the processed data from the front-end. It focuses on estimating the robot’s trajectory and building a map of the environment using optimization or filtering techniques that minimize errors over observations, such as loop closures, when the robot revisits previously mapped areas. The back-end ensures that the SLAM solution is coherent and accurate over time. The following provides a detailed explanation of these two modules.

### 2.1. SLAM Front-End

The front-end module of a SLAM system processes sensor data, typically from cameras or LiDARs, to extract features and estimate the robot’s pose. The two main approaches for front-end visual SLAM (VSLAM) are feature-based and direct SLAM.

#### 2.1.1. Feature-Based SLAM

Underwater VSLAM primarily relies on feature-based methods. These methods focus on identifying and tracking distinctive points or regions of interest (keypoints) within images. Standard feature detectors include scale invariant feature transform (SIFT) [8], speeded up robust features (SURF) [9], and oriented FAST and rotated BRIEF (ORB) [10]. These detectors are robust to variations in illumination, rotation, and scale, making them suitable for underwater environments where lighting conditions can be challenging. The impact of feature point geometrical composition on localization accuracy has been investigated [11]. It showed that selecting features with higher depth variation improves localization accuracy by providing better cues for camera motion estimation.

**Figure 4:**
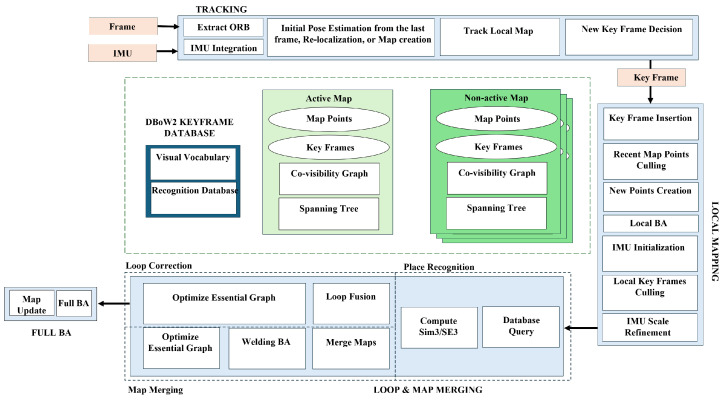
ORB-SLAM3 system architecture illustrating its key components and data flow. The system comprises the tracking module for camera pose estimation using ORB features, the local mapping module to construct and optimize the local map with new keyframes, and the loop closure module to detect loop closures and reduce drift through pose-graph optimization. (Adopted from [12]).

One prominent example of a feature-based SLAM system is ORB-SLAM3 [12]. The architecture and main modules of ORB-SLAM3 are shown in Figure 4. This system offers several key advantages:

- **Visual-inertial Fusion:** ORB-SLAM3 tightly integrates visual data with inertial measurements (e.g., accelerometers and gyroscopes) to achieve robust and accurate pose estimation, particularly beneficial underwater where visual data may be limited.
- **Multi-map SLAM:** The system can manage multiple maps during long periods of limited visibility. When the robot revisits a previously explored area, these maps can be merged, improving overall map accuracy.
- **Global Re-use of Information:** Unlike traditional VO systems that only use recent information, ORB-SLAM3 leverages data from all previously observed keyframes, improving overall system accuracy.

#### 2.1.2. Direct SLAM

Direct SLAM methods, in contrast to feature-based approaches, operate directly on the intensity values of pixels within images. Instead of extracting and tracking features, these methods minimize the photometric error, which refers to the difference in intensity values between corresponding pixels in consecutive images. This approach can be advantageous in environments with low texture or repetitive patterns.

**Figure 5:**
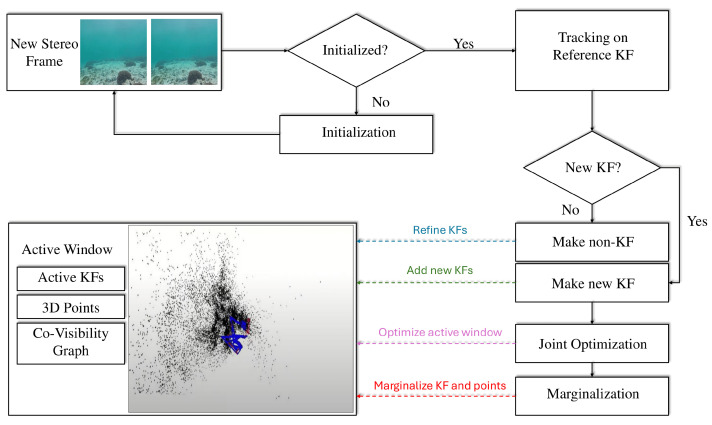
DSO-SLAM system architecture illustrating its main components. DSO is a direct VSLAM system that minimizes photometric error over selected pixels without explicit feature extraction. Key modules include photometric calibration initialization, frame management for keyframe selection and marginalization, and nonlinear optimization for jointly optimizing camera poses and depth parameters. (Adopted from [13]).

An example of a state-of-the-art direct SLAM method is direct sparse odometry (DSO) [13], whose architecture is presented in Figure 5. DSO offers several advantages:

- **Robustness in Featureless Environments:** By directly analyzing pixel intensities, DSO is effective even in environments with few features, which can be common underwater.
- **Full Photometric Calibration:** The method includes calibration for factors such as exposure time, lens vignetting, and non-linear camera responses, improving the accuracy of photometric error minimization.
- **Efficient Pixel Sampling:** DSO uniformly samples pixels across the image, capturing information from regions with sharp edges and smooth intensity variations.

Although both feature-based and direct SLAM methods have advantages and disadvantages, the choice of approach in underwater applications often depends on the specific environment and sensor configuration. Feature-based methods may be preferred when apparent features are present, while direct methods can offer robustness in feature-depleted environments.

### 2.2. SLAM Back-End

SLAM systems estimate the robot’s pose and map the surrounding environment [1]. The challenge lies in optimizing this estimate over time to account for sensor noise and inaccuracies. The SLAM back-end plays a crucial role in performing state optimization. While VO provides short-term pose estimates, errors accumulate over time. The back-end of a SLAM system addresses this issue by enabling state optimization on a larger scale and for longer durations, refining both the robot’s pose and the map. Two main approaches exist for back-end SLAM: filtering-based and optimization-based. Table 1 provides a comparison of these approaches.

#### 2.2.1. Filtering-Based SLAM

Filtering-based SLAM uses a probabilistic framework to represent uncertainties in both the robot’s pose and the map. Various filtering methods are commonly employed in this approach. One widely used method is the extended Kalman filter (EKF) SLAM, which uses a state-space representation to estimate both the robot’s pose and the map’s landmarks. While EKF is computationally efficient, it struggles with non-linearities, which can affect accuracy in more complex environments [14]. The unscented Kalman filter (UKF) SLAM addresses these limitations by applying a deterministic sampling technique, making it better suited for non-linear models.

Another prominent approach is particle filter (PF) SLAM, which represents the robot’s pose and map using particles. PF-based SLAM handles non-linearities effectively but can be computationally expensive in large environments [15]. The Rao-Blackwellized particle filter (RBPF) SLAM combines particle filters for pose with Kalman filters for landmarks, offering an efficient solution.

Several studies have advanced filtering-based SLAM:

- Stachniss et al. [16] presented a grid-based FastSLAM for exploration, enabling active loop closure.
- Grisetti et al. [17] developed an RBPF integrating odometry and laser data for improved sampling.
- Heshmat et al. [18] used camera oscillations to enhance depth estimation.
- Sadeghzadeh-Nokhodberiz et al. [19] proposed an RBPF for quadcopters addressing sensor faults.
- Nie et al. [20] introduced LCPF, an RBPF LiDAR SLAM with loop detection.

#### 2.2.2. Optimization-Based SLAM

Optimization-based SLAM formulates the problem as graph optimization, with poses and landmarks as nodes and measurements as constraints. The goal is to minimize constraint violations for accurate maps and trajectories. A prominent example of this approach is graph-based SLAM, which uses optimization algorithms to identify the most likely configuration of the graph that represents the robot’s trajectory and the environment. An extension of this is factor-graph SLAM, which incorporates sensor noise and uncertainties into the optimization process, refining the system’s robustness.

Key contributions include the following:

- Klein and Murray [21] introduced a real-time tracking system separating tracking and mapping.
- Strasdat [22] showed keyframe bundle adjustment’s superior accuracy.
- Latif et al. [23] proposed a method for robust loop closure detection.
- Li et al. [24] developed an NN-based FastSLAM to reduce errors.
- Bustos et al. [25] used rotation averaging for simplified SLAM.
- Liu et al. [26] presented a bundle adjustment for LiDAR SLAM reducing drift.

In conclusion, the SLAM back-end is critical for refining pose and map accuracy. Filtering-based methods are efficient but may struggle with non-linearities, while optimization-based approaches offer robustness at higher computational cost. The choice depends on application needs. These principles, established in terrestrial robotics, require adaptation for underwater environments due to unique challenges. The next section explores these challenges, introducing a classification of traditional underwater SLAM methods based on UWSNs and the growing importance of UWSNs in advancing the field.

## 3. Underwater SLAM: Background and Overview

Underwater SLAM is a critical technology for enabling AUVs to navigate and map complex, GNSS-denied environments. With applications ranging from marine exploration and environmental monitoring to infrastructure inspection and resource mapping, underwater SLAM plays a pivotal role in advancing underwater robotics. However, the aquatic environment presents unique challenges, including limited visibility, dynamic conditions, sensor noise, and communication constraints, which complicate the deployment of traditional SLAM methods. Over the years, researchers have developed specialized techniques to address these challenges, evolving from early adaptations of terrestrial SLAM to advanced, multi-sensor fusion and deep learning-based approaches. This section provides an overview of the key challenges, the evolution of underwater SLAM, and the growing importance of UWSNs in advancing the field.

### 3.1. Underwater SLAM Challenges

Underwater SLAM faces unique challenges due to the harsh and dynamic nature of aquatic environments. Limited visibility, caused by suspended particles, turbidity, and unpredictable lighting, significantly hinders feature extraction and matching, which are critical for SLAM algorithms [27,28]. Additionally, dynamic elements such as marine life and water currents introduce noise and distortions, further complicating sensor data interpretation [29].

Underwater communication is another major challenge, as acoustic signals suffer from latency, limited bandwidth, and multipath effects, making real-time data exchange difficult [30]. Computational constraints also play a role, as AUVs must operate with limited processing power and energy resources, necessitating efficient algorithms for real-time SLAM [31]. These challenges collectively make underwater SLAM a complex problem, requiring specialized solutions beyond those used in terrestrial environments.

### 3.2. Evolution of Underwater SLAM

**Figure 6:**
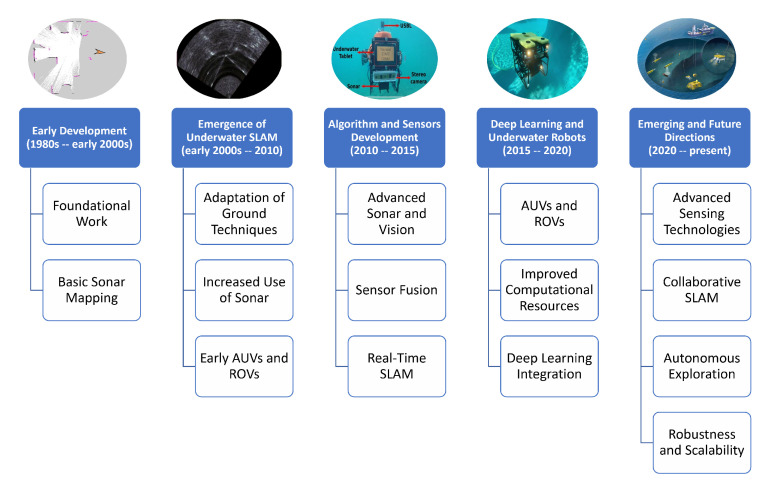
Different stages of underwater SLAM development, from traditional to deep learning-based methods.

Despite substantial advancements in SLAM algorithms, their adaptation for underwater applications has also progressed. Figure 6 highlights the different stages in the progression of underwater SLAM, from traditional geometric approaches to advanced deep learning-based techniques. The early stages of SLAM development before 2000 focused on fundamental concepts and basic implementations, laying the groundwork for future advancements. Between 2000 and 2010, underwater SLAM emerged as a distinct research area, driven by the increasing need for autonomous navigation in marine environments.

From 2010 to 2015, advancements in algorithms and sensor technologies significantly improved the reliability and accuracy of SLAM systems. This period saw the integration of novel sensors, such as acoustic and vision-based systems, tailored to the specific challenges of underwater environments. Between 2015 and 2020, incorporating deep learning and using more sophisticated underwater robots marked a transformative shift in SLAM capabilities. Deep learning enabled more robust feature extraction, data processing, and decision making, significantly enhancing SLAM performance under these challenging conditions.

The present stage, characterized by emerging and future technologies, focuses on leveraging advanced DL technologies, multi-modal sensor fusion, real-time processing, and collaborative systems to address the remaining limitations of underwater SLAM. Advancing SLAM for underwater use is crucial for addressing the unique challenges posed by underwater settings ultimately driving progress in autonomous underwater exploration and sustainable resource management [27].

The evolution of underwater SLAM reflects a transition from traditional methods to advanced, adaptive techniques tailored for aquatic environments. Early SLAM approaches, such as EKF SLAM and graph-based SLAM, were initially developed for terrestrial robots operating in structured environments with abundant features and stable illumination [32,33]. However, these methods often struggled underwater due to low visibility, sensor noise, and dynamic conditions, leading to degraded accuracy and reliability.

To address these limitations, researchers began refining traditional SLAM methods for underwater applications. For instance, multi-sensor fusion techniques incorporating inertial measurement units (IMUs), Doppler velocity logs (DVLs), and acoustic sonars were developed to compensate for unreliable visual data and stabilize pose estimates [34,35]. Filtering-based methods like EKF-SLAM and particle filter-based SLAM were adapted to handle acoustic measurements and nonlinear motion, while optimization-based methods integrated acoustic range constraints and sonar-based loop closures [36].

Bioinspired algorithms also emerged, drawing inspiration from marine life to navigate noisy, low-visibility environments effectively [36]. These advancements enabled SLAM systems to adapt to diverse underwater environments, ranging from structured settings, pipelines, and seabed infrastructure to unstructured or dynamic scenarios, coral reefs, and open water, [37]. Table 2 provides a summary of traditional SLAM methods used in underwater applications, highlighting their sensors, approaches, and limitations.

As underwater SLAM continues to evolve, the role of UWSNs has become increasingly important. UWSNs provide a framework for distributed sensing, communication, and data sharing, addressing many of the challenges associated with underwater environments. The integration of deep learning and UWSNs has enabled more accurate data and effective monitoring of underwater environments [38]. The next subsection explores the significance of UWSNs and their potential to revolutionize underwater SLAM.

**Table 2:** Examples of traditional SLAM methods in underwater environments.

Reference	Sensor	Front-End	Back-End	Focus	Findings	Limitations
Bonin-Font et al. (2015) [1]	Stereo Cameras	-	Graph-SLAM & EKF-SLAM	Localization, Mapping	Graph-SLAM outperforms EKF-SLAM	Limited by imaging conditions
Demim et al. (2022) [39]	Sonar	Hough Transformation	ASVSF SLAM	Localization, Mapping	Improved accuracy compared to EKF-SLAM	Requires further validation
Rahmati et al. (2019) [40]	Generic	SURF	SLAM with Adaptive Sampling	Navigation, Mapping	Efficient data collection in water bodies	Limited by tether dependence
Zhang et al. (2022) [2]	Optical Cameras	ORB Feature Detection	ORB-SLAM2	Localization	Effective for underwater robot localization	Requires distortion correction
Carrasco et al. (2015) [28]	Stereo Cameras	-	Graph-SLAM	Navigation, Localization, Control	Stereo vision improves localization precision	Computationally intensive
Palomeras et al. (2019) [35]	Multi-beam Sonar	ICP Algorithm	Active SLAM	Localization, Mapping	Maintains vehicle uncertainty bounded	Limited by environmental variability

### 3.3. Importance of Underwater Wireless Sensor Networks (UWSNs)

UWSNs have emerged as a critical enabler for advanced underwater SLAM systems. UWSNs facilitate distributed sensing, communication, and data sharing among multiple AUVs and static sensor nodes, enhancing the robustness and scalability of SLAM in large or complex environments [5]. By leveraging UWSNs, SLAM systems can overcome limitations such as limited sensor range and communication constraints, enabling cooperative SLAM where multiple agents collaboratively build and update maps [41].

UWSNs also support the integration of heterogeneous sensor data, such as acoustic, optical, and inertial measurements, improving the accuracy and reliability of SLAM in challenging conditions [42]. Furthermore, UWSNs enable real-time data exchange and processing, which is essential for dynamic environments where rapid adaptation is required [5].

Given the growing importance of UWSNs in underwater SLAM, we propose a new classification framework that categorizes SLAM techniques based on their communication and networking considerations. This framework highlights the role of UWSNs in advancing underwater SLAM and provides a foundation for future research in this area.

## 4. Proposed SLAM Classification Based on UWSNs

The classification of underwater SLAM systems is critical for understanding the capabilities, limitations, and applicability of different approaches across various operational scenarios. They provide a fundamental taxonomy for understanding their design, implementation, and use cases. Traditional classifications of SLAM methods have primarily focused on sensor modalities, computational techniques, and environmental adaptability. While these classifications provide a foundational understanding, they often overlook the critical role of communication and networking in enhancing SLAM performance. This section introduces a novel classification framework for underwater SLAM based on the integration with UWSNs. By emphasizing the role of communication and collaboration, this new classification aims to address the limitations of traditional approaches and provide a more comprehensive understanding of how SLAM systems can be optimized for complex underwater environments.

### 4.1. Traditional SLAM Classifications

**Figure 7:**
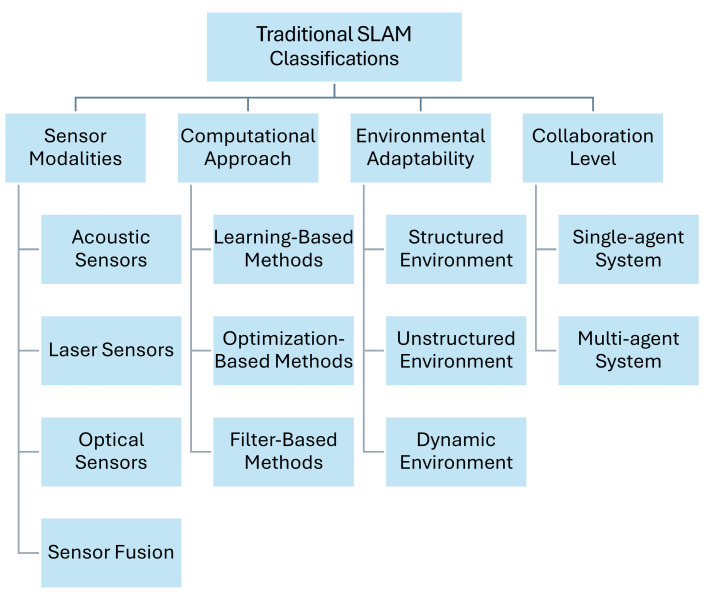
Underwater SLAM classifications based on sensor modalities, computational approaches, environmental adaptability, and collaboration level.

Various classification methods have been proposed to better understand and categorize underwater SLAM techniques. Figure 7 illustrates traditional classifications based on sensor modalities, computational approaches, environmental adaptability, and collaboration levels. These classifications provide a foundational framework for understanding the diverse methodologies employed in underwater SLAM.

#### 4.1.1. Sensor Modalities

Underwater SLAM techniques are often categorized based on the types of sensors deployed. Acoustic sensors, such as sonar, Doppler velocity logs (DVL), and acoustic beacons, are widely used due to their effectiveness in environments where light penetration is limited [43]. Optical sensors, including monocular and stereo cameras, facilitate VSLAM but face challenges due to light scattering and absorption in water [28]. Laser sensors, while providing high-resolution measurements, are constrained by water turbidity and absorption properties [44]. To overcome the limitations of individual sensors, sensor fusion approaches combine multiple sensor types, leveraging the strengths of each modality to improve overall SLAM performance [45].

#### 4.1.2. Computational Approaches

From a computational perspective, SLAM methods can be divided into filter-based, optimization-based, and learning-based techniques. Filter-based methods, such as EKF and particle filters (PF), estimate the system’s state by sequentially updating probabilities, making them suitable for real-time applications [14]. Optimization-based methods, such as graph-based SLAM, refine pose estimates by minimizing localization errors throughout the trajectory, providing more accurate and consistent mapping results [22]. Learning-based methods incorporate machine learning (ML) and DL algorithms to model complex, nonlinear relationships in the data, potentially improving robustness and adaptability in challenging environments [46].

#### 4.1.3. Environmental Adaptability

Environmental adaptability is another critical aspect of classifying SLAM techniques. Systems designed for structured environments, such as pipelines or seabed infrastructure, perform well in settings with geometric regularities that can be exploited for localization and mapping [37]. In contrast, techniques suitable for unstructured environments, such as open water or coral reefs, handle complex terrains that may lack distinctive features, requiring more sophisticated perception algorithms [36]. SLAM systems operating in dynamic environments, where conditions such as water currents and marine life introduce additional noise, must be capable of handling moving objects and changing conditions to ensure reliable performance [29].

#### 4.1.4. Collaboration Levels

The level of collaboration distinguishes between single-agent and multi-agent systems. Single-agent systems involve SLAM performed by a single underwater vehicle, relying solely on its onboard sensors and processing capabilities [31]. Multi-agent systems, on the other hand, involve collaborative SLAM, where multiple robots share information to build a collective map and improve localization accuracy [47]. This collaboration often requires robust communication protocols to handle data exchange between agents, particularly in environments where acoustic communication is limited by bandwidth and latency [30].

### 4.2. New Classification Based on UWSNs

**Figure 8:**
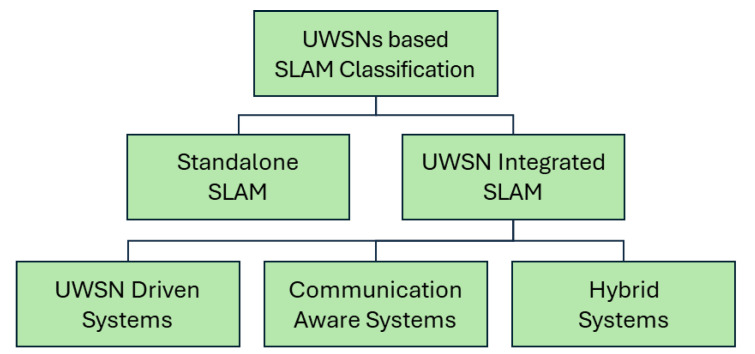
New proposed classifications for underwater SLAM based on UWSN integration.

Classifying SLAM methods specifically for underwater communication is challenging due to the unique constraints of underwater environments. Given the growing reliance on networked underwater exploration and multi-agent collaboration, we propose a new classification based on the integration with UWSNs, as shown in Figure 8. This classification introduces four categories, each addressing different levels of communication and collaboration in underwater SLAM systems.

Underwater SLAM systems increasingly operate in networked environments where communication constraints directly impact performance. Our UWSN-based classification addresses this reality by providing the following:

- Deployment Guidance: matches system capabilities to mission requirements, for example, standalone for deep trench exploration and UWSN-integrated for coastal monitoring;
- Resource Optimization: helps balance the computational load between onboard processing and network utilization;
- Adaptive Design Framework: enables dynamic reconfiguration based on changing channel conditions.

#### 4.2.1. Standalone SLAM Systems

Standalone SLAM systems operate independently without relying on external communication networks. These systems are suitable for environments where communication is limited or unavailable, relying solely on onboard sensors and processing capabilities. However, since real-time data transmission is often infeasible in underwater scenarios, standalone systems must store large volumes of sensor data locally, requiring high-capacity storage solutions. Post-mission, the collected data can be processed in a deferred manner, either when the vehicle resurfaces or through opportunistic communication with ground stations or drones once it establishes a connection. While standalone systems are robust in isolated settings, their performance is constrained by the limitations of individual sensors, the lack of external data inputs, and the challenges of managing extensive onboard data storage [1].

#### 4.2.2. UWSN-Integrated SLAM Systems

UWSN-integrated SLAM systems enhance performance by incorporating external data and communication capabilities through UWSNs. These systems improve localization accuracy and enrich generated maps by leveraging networked sensors and communication. For example, acoustic beacons and distributed sensor nodes can provide additional environmental data, enabling more robust and accurate SLAM solutions [48].

#### 4.2.3. Communication-Aware SLAM Systems

Communication-aware SLAM systems adapt their algorithms based on communication constraints and network conditions. These systems optimize operations by considering factors such as bandwidth limitations, latency, and the reliability of underwater communication channels. By dynamically adjusting data exchange strategies, communication-aware systems can maintain performance even in challenging communication environments [49].

#### 4.2.4. Hybrid SLAM Systems

Hybrid SLAM systems combine standalone and communicative elements, dynamically switching modes based on communication availability. When communication networks are accessible, these systems leverage external data and collaborative opportunities; otherwise, they operate autonomously using onboard resources. This flexibility makes hybrid systems particularly suitable for environments with intermittent communication, such as deep-sea exploration or areas with variable acoustic conditions.

The proposed UWSN-based classification directly addresses key operational challenges in underwater SLAM deployment. Standalone systems enable missions in communication-denied environments. UWSN-integrated systems enhance monitoring capabilities through collaborative sensing. Communication-aware systems optimize bandwidth usage in dynamic channels, reducing data loss through adaptive compression strategies. Hybrid systems provide mission continuity in variable conditions. This framework empowers practitioners to select optimal architectures based on environmental constraints, network availability, and mission requirements.

In summary, various classification schemes—based on sensors, computational methods, environmental factors, and communication integration—further clarify the capabilities and limitations of different approaches. These classifications guide the selection and design of SLAM solutions, ensuring practitioners choose methods aligned with their underwater environment and operational constraints.

## 5. Sensors Driving Underwater SLAM

In the previous section, we classified underwater SLAM methods based on their underlying principles, sensors, and computational approaches. Building on that foundation, we now focus on the sensing technologies that form the backbone of these SLAM systems. Underwater environments pose unique challenges, including the absence of GNSS signals, variable visibility conditions, and the presence of dynamic elements such as marine life or moving particles. Many sensors and modalities have been employed to overcome these obstacles. These range from acoustic sensors that leverage the propagation characteristics of sound waves underwater to optical sensors that capture high-resolution imagery in more apparent conditions to emerging laser-based and multi-sensor fusion approaches. Understanding these sensing technologies’ capabilities, limitations, and suitable applications is critical for developing robust and accurate underwater SLAM solutions.

**Figure 9:**
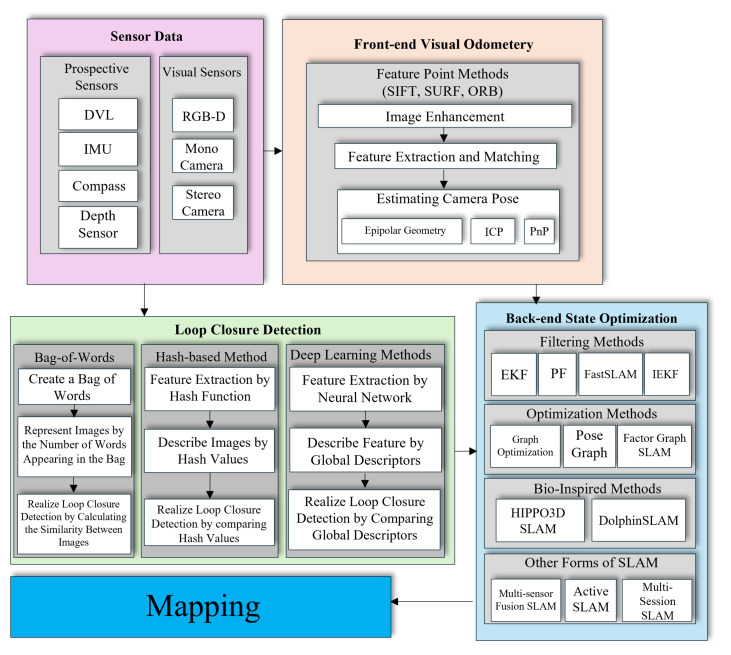
Overview of sensors and methods used in underwater SLAM applications. The figure presents a comprehensive summary of sensors, front-end VO methods, loop closure detection techniques, and back-end state estimation algorithms. This visualization highlights various combinations of sensing technologies and SLAM methodologies that address the challenges of underwater navigation. (Adapted from Zhang et al. [27]).

This section delves into the primary sensing modalities and their roles in underwater SLAM. We begin by examining vision-based sensors, which provide rich visual information essential for detailed mapping but whose performance is often constrained by water turbidity and lighting conditions. We then discuss acoustic sensors, such as sonar, that offer long-range coverage and reliability in low-visibility settings, albeit at lower resolution. We also consider emerging technologies like laser-based sensors that combine high accuracy with underwater-appropriate propagation characteristics. Finally, we explore sensor fusion techniques, which integrate data from multiple sensor types to enhance robustness, improve map quality, and provide a complete understanding of the environment. Figure 9 summarizes the sensors and technologies utilized in each stage of the SLAM algorithm.

By analyzing these sensing modalities’ capabilities, limitations, and applications, we gain insights into how researchers and practitioners can tailor underwater SLAM solutions to specific environmental conditions and mission requirements. Moreover, the integration of machine learning and deep learning methods for sensor data interpretation is steadily improving the adaptability and effectiveness of underwater SLAM systems. Ultimately, selecting the right combination of sensors and processing strategies is key to achieving accurate and efficient navigation and mapping under the challenging conditions of the underwater domain. Table 3 summarizes the advantages, disadvantages, and applications of vision, LiDAR, and sonar sensors for underwater SLAM applications.

In summary, each sensor offers distinct advantages and limitations, and the choice depends on the specific requirements of the application and the environmental conditions.

### 5.1. Vision-Based SLAM

Underwater environments pose serious challenges for SLAM techniques due to limited visibility, varying illumination, and dynamic conditions caused by moving marine life and shifting currents. Vision-based approaches have emerged as a crucial solution, particularly in clear water conditions where visual features can be reliably detected. Their ability to record textures and features makes them ideal for tasks like habitat mapping, underwater archaeology, and marine biology research, where detailed imagery is crucial for understanding the environment [50]. This section examines these methods through three key perspectives: monocular systems, stereo vision approaches, and advanced image enhancement techniques.

#### 5.1.1. Monocular Vision Systems

Monocular camera systems offer a lightweight and cost-effective solution for underwater SLAM, though they face inherent scale ambiguity challenges. Recent advancements have significantly improved their reliability through hybrid visual-inertial approaches. For instance, Ou et al. [51] demonstrated that combining active monocular vision with inertial measurements can reduce scale estimation errors. Integrating camera data with acoustic or inertial measurements (from IMUs or DVLs) helps compensate for the limitations of relying solely on visual input. This fusion of sensor data can offset the impact of bad visibility or changing conditions, leading to more robust and adaptable SLAM solutions. Jung et al. [52], for example, enhanced an AUV’s SLAM performance by supplementing camera feeds with artificial landmarks and additional navigation sensors, resulting in a more stable and reliable SLAM framework. These systems are particularly effective in shallow water inspections where their simplicity and low power consumption provide operational advantages. However, their performance degrades in feature-poor environments or under extreme lighting variations, necessitating careful system design and frequent loop closures to maintain accuracy.

#### 5.1.2. Stereo Vision Approaches

Stereo camera configurations provide direct depth estimation through epipolar geometry, offering more robust 3D reconstruction than monocular systems. The work of Lu et al. [53] showcases this advantage through their ORB-SLAM3-VIP implementation, which achieves precise navigation by fusing stereo depth with IMU data. Stereo systems typically maintain better trajectory estimation at greater depths compared to monocular alternatives. The trade-off comes in computational complexity and hardware requirements—stereo processing demands approximately twice the computational resources of monocular systems while also requiring careful calibration to maintain accuracy in varying water conditions.

#### 5.1.3. Image Enhancement Techniques

Deep learning-based image enhancement has revolutionized underwater visual SLAM by addressing fundamental visibility challenges. These techniques specifically target key problems: turbidity compensation, low-light enhancement, and real-time processing. Liu et al. [54] developed adaptive filtering methods that improve feature-matching accuracy. Modern implementations balance enhancement quality with computational efficiency [55]. These advancements have expanded the operational envelope of vision-based SLAM to previously challenging environments, though they still face limitations in extreme turbidity or complete darkness.

As research continues, these integrated strategies—improved image processing, sensor fusion, and continuous real-time mapping—make vision-based SLAM increasingly viable across various underwater scenarios. Whether used for detailed inspections, ecological surveys, or the exploration of wreck sites, vision-based SLAM systems are steadily evolving to meet the complex demands of underwater environments. For further insights into current methods, findings, and challenges, Table 4 summarizes recent studies in this field.

### 5.2. Acoustic-Based SLAM

Underwater environments often challenge visual sensors due to low visibility, limited light, and suspended particles that scatter or absorb light. In such conditions, acoustic sensors, especially sonar systems, provide a reliable alternative for SLAM tasks. Unlike optical methods, which depend on clarity and lighting, acoustic waves propagate efficiently in water, allowing sonar-based approaches to work effectively in murky, deep, or low-visibility environments [34].

**Figure 10:**
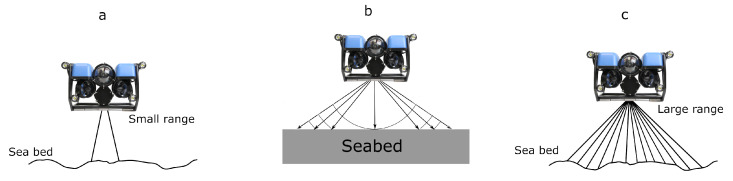
Typical sonar: (**a**) single-beam sonar; (**b**) side-scan sonar; (**c**) multibeam sonar adopted from [57].

Acoustic SLAM leverages sonar signals—ranging from single-beam and side-scan to multi-beam configurations—to navigate and map underwater areas (see Figure 10). Because acoustic waves can travel long distances with minimal attenuation, sonar-based methods are particularly valuable for large-scale mapping, deep-sea exploration, underwater infrastructure inspection, and search and rescue operations. They remain robust when visual cues are absent or severely diminished, offering a distinct advantage over vision-based approaches [43].

However, acoustic systems are not without their challenges. Sonar data can suffer from noise interference, and their spatial resolution generally lags behind that of high-quality optical sensors. Consequently, acoustic SLAM maps may lack the fine detail provided by vision-based methods. Additionally, acoustic noise, multi-path reflections, and complex signal processing requirements can complicate data interpretation, increasing the operational complexity and cost of deploying and maintaining these systems [58].

Recent advancements have significantly enhanced the effectiveness of acoustic SLAM, addressing challenges like poor bearing accuracy and resolution. Researchers have adapted algorithms such as FastSLAM variants to better process sonar data, while techniques like CNN-based sonar image matching have achieved superior accuracy compared to classical methods [59,60]. Low-cost forward-looking sonar systems have demonstrated feasibility for navigation and feature reacquisition using innovative SLAM approaches like pose-graph optimization [61]. Similarly, enhanced loop closure detection methods utilizing acoustic image segmentation and graph-based models have improved mapping robustness in real-world scenarios [62]. Filter-based methodologies, such as RBPF-SLAM, effectively manage data-intensive sonar environments, while YOLOv7 applications to 3D reconstruction further refine state estimation and mapping accuracy [63,64].

The availability of dedicated datasets, including mechanical scanning sonar (MSS) data with ground truth localization, has accelerated research in underwater SLAM [65]. Forward-looking sonar has also shown great promise, utilizing factor graph optimization with techniques like SO-CFAR and adaptive thresholding (ADT) for noise filtering and WICP algorithms for feature registration, achieving an 8.52% improvement in RMSE over dead reckoning [66]. As acoustic sensor technologies and SLAM algorithms evolve, these systems address key challenges like data fidelity, real-time processing, and large-area coverage. Table 5 summarizes recent developments, highlighting methods, findings, and limitations driving innovation in acoustic-based underwater SLAM.

In summary, acoustic-based SLAM is a robust solution for underwater applications where environmental conditions hinder optical methods. While acoustic sensors may produce less detailed maps and require specialized expertise, their resilience under challenging environments underscores their importance for long-range communication, extensive mapping, and reliable navigation. With ongoing improvements in sensor technology, data processing, and algorithmic approaches, acoustic SLAM is poised to play an increasingly vital role in underwater exploration and robotics.

### 5.3. Laser-Based SLAM

Laser sensors are an emerging technology in underwater SLAM, offering a novel approach through laser-based acoustic generation and detection. These sensors are particularly valuable for underwater applications as they combine laser measurements’ high accuracy with acoustic waves’ propagation advantages. Fibre laser-based sensors, for example, provide robust solutions for underwater surveillance, offering lightweight and deployable options that enhance mapping capabilities. Such sensors are increasingly used underwater for high-resolution mapping and loop closure [44,69].

### 5.4. Multi-Modal-Based SLAM

Underwater environments often limit the effectiveness of single-sensor SLAM methods. Clear visibility may favor vision-based systems, but murky or low-light conditions can render optical sensors ineffective. Acoustic sensors, while robust in turbid waters, may struggle with fine details or rapidly changing scenes. LiDAR may provide precise distance measurements, but it can be challenged by aggressive vehicle motion or environments lacking distinctive features. To overcome these inherent trade-offs, researchers have increasingly turned to sensor fusion—also known as multi-modal SLAM—which integrates data from multiple sensor types to leverage their complementary strengths [43].

Multi-modal SLAM systems combine information from various modalities—such as cameras, sonar, LiDAR, IMUs, DVLs, and acoustic positioning systems like USBL—to produce more robust and comprehensive maps. By blending these diverse inputs, multi-modal SLAM can compensate for the weaknesses of each individual sensor. For example, pairing optical cameras with multibeam sonar improves 3D reconstruction and mapping accuracy in areas where vision alone would struggle [70]. Integrating inertial data helps stabilize pose estimates when visual or acoustic features are sparse, while LiDAR data can enhance detail and precision under challenging lighting conditions.

Learning-based approaches have significantly advanced sensor fusion quality in underwater robotics. CNNs-based techniques enhance sonar imagery and leverage sensor complementarity, enabling more effective underwater perception and navigation [71]. Visual-inertial systems have also been adapted for underwater environments, integrating visual and inertial data to maintain reliable tracking even in challenging conditions such as fluctuating illumination and sparse features. For example, systems like USBL-aided navigation incorporate multiple sensory inputs—including VO and inertial measurements—to improve trajectory estimation [72]. Extensions to existing visual-inertial state estimation frameworks, such as integrating acoustic range data, have proven effective for reconstructing underwater structures in complex scenarios like caves and shipwrecks [73]. Furthermore, tightly coupled SLAM systems such as SVIn2 fuse sonar, visual, inertial, and water-pressure data, achieving robust initialization, loop closing, and localization under harsh underwater conditions, including haze, low light, and motion blur [74]. These innovations highlight the potential of sensor fusion to overcome the unique challenges of underwater environments.

S. Ma et al. introduce a novel tightly coupled monocular-inertial-pressure (IP) sensor fusion method tailored for the underwater localization of a biomimetic robotic manta. Building on ORB-SLAM3 monocular visual-inertial odometry, depth measurements from a pressure sensor are incorporated, and a two-step monocular initialization strategy—first using visual-pressure (VP) measurements and then constructing inertial pressure depth residuals—significantly improves scale estimation. Following successful initialization, a visual-inertial-pressure (VIP) joint optimization enhances both position and attitude estimates, offering valuable insights for robust underwater localization of biomimetic robotic platform [75].

Y. Huang et al. explore advanced sensor fusion techniques that address scale drift and stability issues under variable lighting, turbidity, and acoustic conditions. By employing innovative data association strategies and refined sensor integration, this work provides further evidence that combining multiple sensors and careful calibration can yield more reliable navigation solutions across diverse underwater scenarios [76].

Multi-modal SLAM also benefits specialized applications such as underwater infrastructure inspection and ecological surveys. Combining sonar for large-area coverage, cameras for detailed imagery, and inertial sensors for stability results in more accurate and adaptable SLAM solutions. For instance, fusing stereo vision and multi-beam sonar can improve feature tracking [45], while visual-LiDAR approaches help overcome aggressive motion and poor lighting conditions [77]. Unmanned surface vehicles equipped with multiple sensors have demonstrated the capability to produce detailed above-and-below-water maps [78].

Nevertheless, multi-modal SLAM introduces challenges. Integrating different sensors requires complex algorithms, high computational resources, and careful real-time data processing. Calibrating and synchronizing heterogeneous sensors add operational complexity, and interpreting fused data demands specialized expertise. Despite these difficulties, multi-modal SLAM holds significant promise. By drawing on multiple sensors and leveraging refined initialization strategies (as in [75]) or advanced data association methods (as in [76]), SLAM systems can better adapt to low visibility, fluctuating conditions, and extended missions. Table 6 provides a concise overview of recent advancements and methodologies in sensor fusion for SLAM systems. It summarizes key research contributions, highlighting the problems addressed, methods employed, key findings, and limitations of various sensor fusion approaches

In complex underwater environments, no single sensor modality is sufficient to address all challenges. Adaptive sensor fusion techniques have emerged as a key approach to overcoming these limitations by dynamically combining data from multiple sensors, such as acoustic, optical, and inertial systems. For instance, DVLs provide accurate velocity estimates in feature-depleted regions, while vision-based systems excel in areas with sufficient texture and lighting. When fused, these systems can compensate for each other’s weaknesses, ensuring reliable localization and mapping under diverse conditions. Advanced fusion algorithms also incorporate real-time environmental feedback, enabling context-aware sensor prioritization. For example, in turbid or low-visibility waters, acoustic sensors dominate, whereas optical sensors take precedence in clearer conditions. This adaptability not only enhances SLAM performance but also reduces computational overhead by focusing processing resources on the most reliable sensor data. Such innovations are critical for enabling robust and efficient SLAM in unpredictable underwater scenarios.

## 6. Practical Challenges in Underwater SLAM

Underwater SLAM is a critical technology for enabling autonomous navigation and exploration in subaquatic environments. Unlike terrestrial and aerial SLAM—where communication infrastructure, computational resources, and power supply are relatively stable—the unique and often harsh conditions of underwater settings introduce a range of practical challenges that must be addressed to ensure reliable and efficient SLAM performance. These challenges span multiple domains, including communication, computational resource management, and energy efficiency, each significantly impacting the feasibility and effectiveness of underwater SLAM systems. This section delves into these key challenges, exploring their implications, surveying current solutions, and highlighting ongoing limitations.

### 6.1. Underwater Communication

Underwater data transmission commonly uses four main methods: acoustic waves, optical communication, magnetic induction (MI), and radio-frequency (RF) methods [84,85]. Each modality operates within specific frequency bands and ranges, with trade-offs between bandwidth, latency, and environmental adaptability. Table 7 summarizes these characteristics, including sonar frequencies for SLAM and vision-based systems in varying water conditions.

#### 6.1.1. Acoustic Communication

Acoustic methods are the most widely adopted for AUV navigation and data exchange due to their long-range propagation (hundreds of meters to kilometers) [86]. Typical SLAM systems use sonar frequencies in the following ranges:

- Low-frequency (LF): 1–10 kHz (long-range, ∼10–100 km, low bandwidth);
- Medium-frequency (MF): 10–100 kHz (mid-range, ∼1–10 km, moderate bandwidth);
- High-frequency (HF): 100–500 kHz (short-range, ∼100–1000 m, high resolution for imaging).

Acoustic SLAM systems, for example, side-scan or multi-beam sonars, often operate in the MF/HF bands to balance resolution and range, though performance degrades in shallow water due to multipath interference [35].

#### 6.1.2. Optical Communication

Optical systems achieve high data rates (up to Gbps), making them attractive for SLAM applications that require streaming video to topside stations or other AUVs [87,88]. However, optical systems are limited by water turbidity and require line-of-sight [89]. Vision-based SLAM performance varies with light penetration:

- Clear water: Blue/green light (450–550 nm) penetrates up to 100 m, enabling monocular/stereo SLAM;
- Turbid water: Red light (600–700 nm) is absorbed quickly, necessitating active illumination or acoustic supplements [28].

#### 6.1.3. Electromagnetic Spectrum

- RF: limited to very low frequencies (<30 Hz) for long-range underwater use, with impractical antenna sizes for AUVs [90];
- MI: short-range (<20 m), suitable for localized swarm coordination [91].

Optimizing AUV trajectories in the presence of ocean currents can significantly reduce energy consumption and mission time, particularly in large-scale underwater sensor networks where visible light communication is used for high-rate data retrieval [92]. Additionally, efficient algorithms for multi-AUV placement can maximize sensor coverage and ensure optimal association between sensors and AUVs, even in scenarios with varying sensor priorities [93]. These advancements highlight the importance of integrating communication and motion planning to enhance the performance of optical underwater networks.

In summary, restricted bandwidth, intermittent connectivity, and high latency undermine real-time cooperative SLAM. As an interim solution, many AUVs rely on acoustic links for basic telemetry while performing most SLAM computations onboard. Future hybrid strategies—combining acoustic, optical, and potentially, electromagnetic methods—could enable more flexible data-sharing frameworks in underwater SLAMs.

### 6.2. Computational Resources

Underwater SLAM systems require real-time processing for obstacle avoidance, precise navigation, and stable control in dynamic and unpredictable underwater conditions. Unlike surface or aerial platforms with greater power and cooling capabilities, underwater AUVs typically have limited CPU/GPU resources and battery lifespans. Deep learning-based underwater SLAM algorithms introduce significant challenges due to their computational demands. For instance, underwater images suffer from distortion, turbidity, and variable lighting, requiring extensive processing for meaningful feature extraction [94]. Moreover, modern deep SLAM models, using convolutional, recurrent, and transformer-based architectures with millions of parameters, are highly computationally demanding, especially when fusing multi-modal data [95].

Consequently, real-time inference demands efficient model optimizations, while the high memory and bandwidth requirements of deep learning models challenge resource-limited underwater embedded systems. This necessitates the development of lightweight architectures [96] and the use of techniques like pruning, quantization, and knowledge distillation (KD) [97].

#### 6.2.1. Model Quantization, Pruning, and Knowledge Distillation

DL model compression techniques are crucial for hardware-constrained environments. For instance, model quantization is a compression method that reduces the memory footprint, computational cost, and power consumption of deep learning models by converting high-precision weights and activations into lower-precision formats [98]. In the context of SLAM, a quantized self-supervised local feature approach has been introduced by Li et al. [99] for indirect VSLAM, using an orthogonal transformation to improve feature efficiency. Moreover, pruning can be used in conjunction with quantization in SLAM to remove less important parameters, such as weights, neurons, or layers, without significantly affecting performance [100].

Another compression technique is KD, where a smaller model (student) is trained to replicate the behavior of a larger, more complex model (teacher), improving efficiency while maintaining accuracy. In semantic SLAM, KD has been used to enhance real-time performance in dynamic environments [101]. For instance, a multi-level KD approach has been proposed by Chen et al. [102] to create a lightweight segmentation model, allowing an independent semantic segmentation thread that processes only keyframes, reducing delays. Additionally, a static semantic keyframe selection strategy was proposed for underwater VSLAM by Yang et al. [103] to minimize the impact of dynamic objects, while dynamic probability propagation further refines pose optimization.

#### 6.2.2. Distributed Systems and Edge Computing

Distributed multi-robot clustering systems provide scalability and faster processing speed, making them well-suited for tasks like collaborative mapping and cooperative navigation [95]. One approach by Qi et al. [104] introduces a homogeneous distributed collaborative mapping system using bathymetric cooperative active SLAM, where a server vehicle optimizes positioning accuracy through online path planning based on Fisher information matrix (FIM) metrics. A novel prediction method for inter-vehicle loop closure factors and an augmented matrix determinant lemma reduce computational overhead, improving both accuracy and efficiency in semi-physical simulations. Similarly, to address trajectory drift in AUVs caused by ocean currents, a multi-AUV cooperative navigation algorithm based on a factor graph with stretching nodes’ strategy has been developed by Ben et al. [105]. By introducing ocean current velocities as variable nodes and transforming the FG into a cycle-free structure, this method enhances localization accuracy and stability while maintaining computational feasibility.

Beyond navigation, distributed systems also play a crucial role in edge computing within underwater environments. Underwater IoT relies on AUVs to supplement the limited computational resources of sensor cluster heads. A proposed AUV-aided offloading framework by Chen et al. [106] integrates multiple AUVs, buoys, and low Earth orbit satellites under an edge intelligence service platform, which manages computational resources dynamically.

### 6.3. Energy Efficiency

Underwater robots often operate far from any direct power source, making energy efficiency a key requirement for extended missions. Propulsion already consumes a substantial share of available power; onboard SLAM computations add further strain [29]. Cameras, sonar arrays, and high-power illumination also contribute to the overall energy budget [107]. As a result, frequent battery recharges or replacements become logistically and economically challenging, particularly in remote or deep-sea environments.

Achieving high SLAM accuracy often involves running computationally expensive models at higher frame rates or resolutions [108]. However, each additional network parameter or sensor input can significantly increase power consumption. Techniques like dynamic frame rate adaptation, where the SLAM process lowers frame capture rate in less complex areas, can reduce energy usage while maintaining adequate map quality [42]. Similarly, adopting specialized low-power hardware such as FPGAs or efficient GPU cores can sustain more advanced DL models within the same energy budget.

Some recent research aims to integrate energy considerations directly into the SLAM loop, dynamically balancing exploration and revisiting tasks [109]. By incorporating energy models that account for sensor usage, processor clock speeds, and propulsion, the SLAM system can decide when to switch sensors on/off or how aggressively to update the map. In multi-AUV missions, coordinating battery levels and assigning tasks based on remaining energy further extends mission duration [110].

One promising energy-aware method is dynamic sensor scheduling, which involves selectively activating or deactivating sensors based on environmental conditions and mission requirements. For example, in underwater environments with turbidity or low light, the SLAM system can prioritize sonar data over high-resolution visual inputs, reducing the energy consumed by power-intensive cameras. Conversely, in lit conditions, visual sensors can take precedence to enhance mapping accuracy. Another approach is to adjust the sampling rate of sensors dynamically: Lowering the frame rate of cameras when the robot is stationary or navigating well-mapped areas can significantly cut power usage without sacrificing map quality. Existing work, such as reinforcement learning-based sensor management, offers a framework for learning optimal activation policies that balance energy efficiency and localization performance [111]. These techniques are particularly valuable in underwater SLAM, where variable conditions demand adaptive sensor use.

Another key energy-aware strategy is adaptive DL model compression, which adjusts the complexity of deep learning models in real time to optimize energy consumption while preserving accuracy. Techniques such as model pruning, removing less critical network parameters, or quantization, reducing the precision of model weights, can be applied dynamically based on the current energy budget or computational load. For instance, during routine mapping in familiar areas, a lightweight, pruned model can suffice, whereas a full model might be activated for challenging tasks like loop closure detection in uncharted regions. A multi-model approach could also be employed, where the SLAM system switches between a library of pre-trained models of varying complexity depending on the task or remaining battery level. Frameworks like once-for-all [112] enable such dynamic model selection by training a single supernet from which sub-models can be extracted efficiently, adapting to resource constraints on the fly. These methods reduce the computational burden of DL-based SLAM, making them better suited for energy-constrained underwater missions.

While compression techniques like pruning or KD reduce model size, energy efficiency can also benefit from hardware improvements and optimized scheduling policies that dynamically turn off non-critical components. Overall, ensuring robust underwater SLAM performance requires tackling high power consumption at both algorithmic and system levels. As missions expand in duration and scope, energy-aware methods will be crucial to sustaining effective mapping and navigation without frequent manual intervention.

## 7. Recent Advances in Deep Learning-Based SLAM

Underwater SLAM has traditionally relied on geometrical methods for accurate pose estimation and mapping. However, underwater environments’ complex and dynamic nature presents significant challenges to traditional approaches, including limited visibility, sensor noise, and environmental distortions. DL offers a data-driven alternative, providing robust solutions to these challenges by leveraging large datasets and powerful computational models. This section explores the application of DL in underwater SLAM, focusing on key areas such as feature extraction, pose estimation, loop closure detection, and mapping. We also discuss the challenges unique to underwater environments and how DL techniques address them. DL offers a data-driven alternative to conventional localization and mapping methods.

Comprehensive surveys have explored the effectiveness of deep learning-based VO on global relocalization and SLAM, synthesizing research from robotics, computer vision, and ML to guide future directions. These surveys conclude that the capability of deep learning models to draw from previous experiences and effectively harness new data allows these models to self-learn and adapt to changing environments. [113,114]. This is particularly important in underwater settings, where the scenery is continuously altered and distorted by varying light conditions and other environmental factors in a dynamic aquatic environment.

DL-based underwater SLAM methods, summarized in Table 8, illustrate the substantial impact of DL on improving SLAM techniques, especially in challenging underwater environments. The table presents various methods and findings, highlighting how DL approaches enhance feature extraction, robustness, and real-time performance in SLAM systems.

**Key Trends in DL-Based Underwater SLAM:** The surveyed deep learning approaches reveal several important developments in underwater SLAM. First, architectural evolution is evident, progressing from basic CNNs to more sophisticated designs like Siamese networks and variational autoencoders, yielding accuracy improvements of 30–40% in feature matching and loop closure tasks. Second, we observe a clear shift from supervised methods requiring labeled datasets to unsupervised [115] and self-supervised approaches, addressing the scarcity of annotated underwater data. Third, while early work focused on single modalities (visual or sonar), recent studies demonstrate improved robustness through multi-modal fusion, with Wang’s 2022 VAE achieving 92.31% recall in challenging conditions. However, three persistent limitations include scalability issues with large-scale environments, limited real-world validation, and modality-specific constraints where visual methods struggle with turbidity and sonar approaches lack precision. The most promising direction appears in hybrid systems combining the precision of visual SLAM with the reliability of acoustic sensing through learned fusion mechanisms.

### 7.1. Underwater Feature Extraction for SLAM

Unlike terrestrial environments, extracting meaningful features from underwater scenes presents significant challenges for VSLAM due to low-light conditions, color distortions, blurring, and unreliable keypoints. To address these challenges, recent research leverages ML. One prominent approach involves utilizing supervised learning to identify high-level structural features, enabling AUVs to effectively relocalize within a SLAM graph [123].

Traditional feature extraction methods, such as SIFT, SURF, and ORB which are widely used in terrestrial SLAM, struggle in underwater environments. These handcrafted feature detectors rely on gradient-based keypoints that become unreliable in turbid or noisy conditions, often resulting in low feature matching accuracy. In contrast, DL methods learn robust, data-driven features from large, diverse datasets. This improvement stems from the ability to adapt to varying visibility and lighting through training, offering superior generalization compared to the rigid, predefined rules of traditional techniques. However, DL approaches require substantial computational resources and annotated training data, presenting trade-offs that traditional methods avoid despite their lower performance.

**Figure 11:**
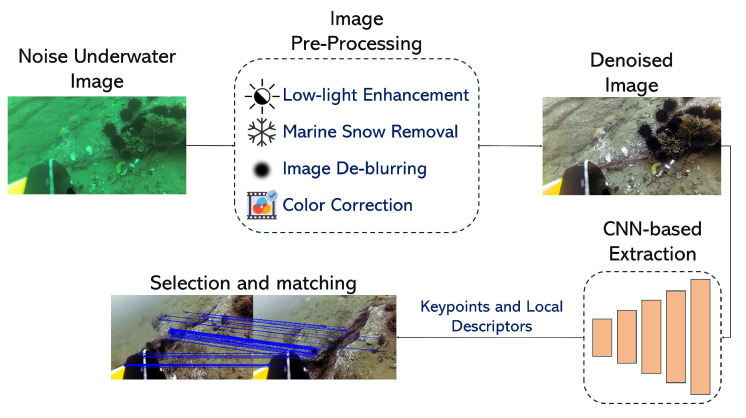
Typical pipeline for feature extraction in DL-based underwater SLAM. The process includes pre-processing to remove marine snow [126], enhance light conditions [127], and reduce underwater noise such as color distortions and blurring [128]. Keypoints are then extracted using CNNs, filtered, and matched. (Adapted from Zheng et al. [129]).

Several other studies demonstrate the effectiveness of DL in underwater feature extraction for robot navigation, as shown in Figure 11. These methods mostly use CNNs to learn compact representations. Maldonado-Ramirez et al. [124] employs convolutional autoencoders to extract salient landmarks from underwater images, improving precision and inference time for underwater SLAM. The findings show improved performance in terms of precision and inference time. Similarly, Peng et al. [125] propose PointNet, a multi-layer perceptron network that receives selected keypoints from a K-nearest neighbor algorithm to extract relevant features.

Reliable feature extraction also requires addressing uncertainty and keypoint selection. A PointNet-based approach for uncertainty estimation in point cloud registration, called PointNetKL [116], has been proposed to address this, offering a computationally efficient alternative to traditional Monte Carlo methods. The method utilizes a neural network to produce the covariance matrix through the parameter estimation of Cholesky Decomposition. Additionally, a CNN-based method [117] has been developed to handle unreliable keypoints caused by shallow water caustics and dynamic objects. This method filters out unreliable points within a VSLAM framework, enhancing robustness. The MARESye system [130] exemplifies how dense 3D data can be captured in visually challenging underwater environments through active and passive imaging. Multi-modal SLAM frameworks thus enhance overall reliability, making them invaluable for underwater exploration and infrastructure inspections.

DL has also been utilized to apply image enhancement for robust feature extraction in low-light, blurry, and noisy underwater images. For example, a CNN-based end-to-end network [127] was developed to tackle low-light environments, incorporating a self-supervised feature point detector and descriptor that enables VSLAM to operate in low-light conditions without requiring paired training data. Similarly, Wang et al. [128] propose a robust DL-based VSLAM system featuring UWNet, a powerful feature generator that extracts accurate keypoints and utilizes knowledge distillation for training. Integrated with ORB-SLAM3, the system demonstrates high precision and robustness in public and self-collected datasets, significantly improving performance in complex underwater scenarios. Generative adversarial networks (GANs) are also used for underwater image enhancement, further improving the performance of SLAM systems in challenging underwater conditions [129]. For nullifying the effect of marine snow noise, Hodne et al. [126] develop two efficient classifiers that run on top of arbitrary keypoint detectors to classify marine snow and subsequently reject it before feature extraction.

Furthermore, CNNs can extract features from fused sonar and camera images, improving perception, obstacle avoidance, and environmental mapping. For instance, UAFMFDet [71] is a dual-branch CNN for acoustic-optical fusion-based object detection, which showed significant improvement compared to other object detection methods. Moreover, sonars can also be utilized for underwater dynamic-SLAM to handle dynamic objects, significantly improving SLAM’s capabilities in underwater contexts, as shown in [131]. The utilized method leverages YOLOv3 in conjunction with a multi-beam sonar for underwater dynamic tracking.

In summary, feature extraction for underwater SLAM is an active research area. With advances in DL, uncertainty estimation, and keypoint selection methods, researchers are developing innovative solutions to address the unique challenges of underwater environments.

### 7.2. Pose Estimation for Underwater SLAM

Accurate pose estimation, the process of determining an underwater vehicle’s 3D position and orientation, is vital for underwater SLAM. This section explores recent advances in DL-based pose estimation methods, significantly improving underwater SLAM performance.

Hou et al. [132] introduce the AMB-SLAM online algorithm for underwater localization in featureless seabeds using acoustic and magnetic beacons. AMB-SLAM utilizes dense neural networks to map between beacon positions and the vehicle position. Another approach proposed by Risholm et al. [133] leverages an EfficientNet CNN feature extractor with bi-directional feature pyramid network for identifying Aruco markers and subsequently obtaining the vehicle’s position.

While dense neural networks are reliable in some applications, they do not factor in the temporal aspect of the input information. Hence, recurrent neural networks (RNN) have proven their reliability in localization since they take into account the time series information of the input inertial, visual, and other sensor data. Specifically, long short-term memory (LSTM) networks have proven their superiority in underwater dead reckoning navigation [134], while gated recurrent unit (GRU) networks have shown enhanced performance as particle filters for underwater target state estimation [135].

**Figure 12:**
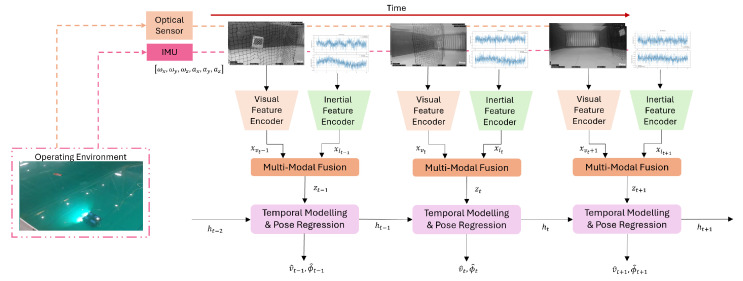
Pipeline for DL-based pose estimation, including encoding visual and inertial features through convolutions and processing the temporal data through LSTMs. (Adapted from Sudevan et al. [137]).

Research work by Teixeira et al. [136] leverages a CNN-LSTM network for underwater pose estimation utilizing a single frame at a time. Similarly, Sudevan et al. [137] evaluate the performance of visual-selective visual-inertial odometry (VS-VIO), a hybrid learning-based multimodal pose estimation framework shown in Figure 12, in underwater environments characterized by low lighting and high turbidity. Unlike in previous work, the proposed network feeds multiple image sequences to the CNN-LSTM network at a time. When testing on the AQUALOC dataset, findings indicate that VS-VIO can dynamically reduce visual modality usage while maintaining accuracy. More recently, the attention mechanism has emerged as a powerful tool for sequence processing. Research by Li et al. [138] showed that a CNN-attention network to process an underwater MEMS IMU sensor can significantly reduce the overall trajectory error.

Moreover, recent research has aimed to utilize DL with DVL sensors for more accurate pose estimates. For instance, BeamsNet [139] is a neural network of dense 1-dimensional convolutions that combines extracted features a gyroscope, and an accelerometer with the DVL readings. Similarly, Topini et al. [140] evaluate different network architectures, including 1-dimensional convolutions, LSTM, dense layers, and Conv-LSTM layers for vehicle velocity estimations in case of temporary DVL failure.

These advancements highlight the growing capability of CNNs when utilized along with recurrent networks for accurate pose estimation in underwater SLAM.

### 7.3. Loop Closure Detection for Underwater SLAM

Loop closure detection, which identifies revisits to previously explored locations, plays a critical role in underwater SLAM for real-time navigation and mapping. It ensures the accuracy of SLAM systems by correcting accumulated drift in position estimates and is essential for creating consistent and reliable maps.

Li et al. [120] present a novel real-time pose-graph SLAM algorithm tailored for underwater ship hull inspections, utilizing a forward-looking sonar to challenges in acoustic underwater SLAM. The algorithm employs a CNN for saliency detection based on the sensitivity of learned global features, followed by both saliency-aware loop closure proposals and robust data association. Furthermore, Bonin-Font et al. [141] introduce a novel global image descriptor named net hash-based loop closure (NetHALOC), trained using a simple CNN.

**Figure 13:**
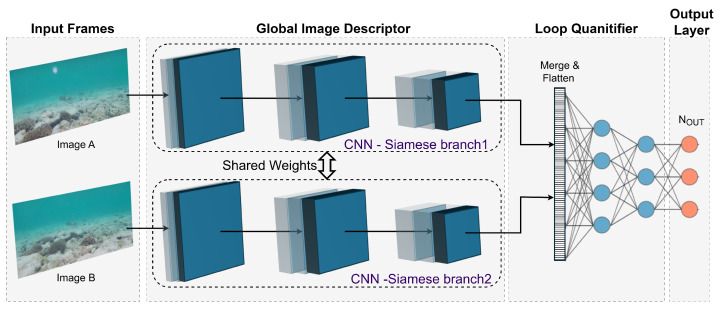
Siamese CNN for loop closure. (Adapted from Burguera et al. [119]).

More recently, Siamese neural networks have emerged as powerful tools for underwater loop closure applications. As demonstrated by Burguera et al. [119], visual loop detection (VLD) can be performed within an underwater VSLAM framework by utilizing convolutional Siamese networks, as shown in Figure 13. This is carried out by passing two underwater images, each to a separate branch of the Siamese network, where pairs that do not close the loop are rejected. Similarly, Tan et al. [122] employ Siamese networks for loop closure in bathymetric point clouds, which is particularly challenging due to the limited presence of distinguishable landmarks on the seabed and the significant drift inherent in dead-reckoning navigation. Moreover, PLNet [125] also utilizes shared-weight multi-branch 3D convolutions with self-attention for matching localization.

Addressing the complexities of dynamic underwater environments, Wang et al. [121] propose a novel loop closure detection method using a variational autoencoder network with a dual branch on the encoder side. This unsupervised approach avoids extensive data labeling and incorporates a semantic object segmentation module to handle fast-moving objects in the underwater environment.

### 7.4. Mapping and 3D Reconstruction for Underwater SLAM

Accurate mapping of underwater environments is crucial for various applications, such as navigation and exploration. This section explores recent advances in underwater mapping using SLAM. Wang et al. [142] survey this rapidly growing field, dividing the work into four key areas: 3D reconstruction from binocular cameras, reconstruction from multiple images, object-focused reconstruction with relaxed calibration requirements, and SLAM-based techniques.

For 3D maps, DL-based underwater monocular depth estimation methods have emerged recently for accurate mapping without the need to utilize stereo cameras or other sensors [143,144]. For instance, some methods utilize domain knowledge to utilize image formation characteristics for synthetic underwater depth map creation [145,146]. Marques et al. [118] introduce a DL-based SLAM method for estimating 3D underwater environments from single video frames. This self-supervised approach leverages a novel depth map prior based on GANs to enhance depth prediction. Beyond monocular cameras, sonars have also proven to be a cheap alternative for 2D mapping tasks [147].

**Figure 14:**
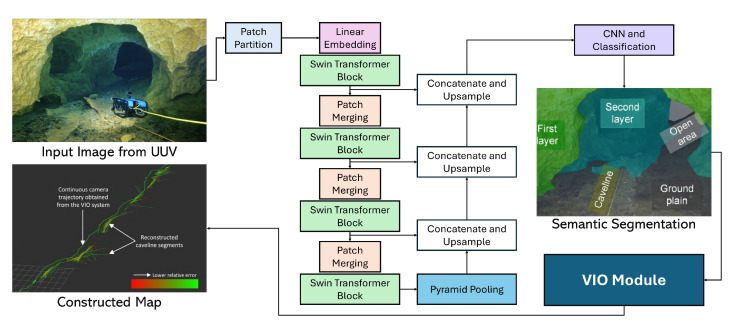
CaveSeg network architecture for semantic mapping in underwater environments. (Adapted from Burguera et al. [149]).

Semantic mapping has recently gained significant traction in underwater SLAM. For instance, Li et al. [148] propose a system combining a spatiotemporal deep neural network for semantic segmentation with a SLAM algorithm to create 3D point cloud maps annotated with semantic labels. Similarly, Abdullah et al. [149] introduce CaveSeg, a four-stage SwinTransformer network for semantic segmentation of underwater cave environments, as illustrated in Figure 14. This method, when paired with visual-inertial odometry (VIO), demonstrated accurate map reconstruction with minimal inference time. These advancements in DL-based mapping highlight innovative methods for generating accurate and detailed representations of underwater environments using SLAM techniques and multi-sensor solutions.

Recent progress in deep learning emphasizes the development of adaptive models tailored for underwater applications. Transfer learning and domain adaptation techniques minimize the need for large, labeled underwater datasets by leveraging pre-trained CNNs that can be fine-tuned on limited underwater imagery. This significantly enhances feature extraction accuracy in turbid conditions. Additionally, GANs have been employed for underwater image enhancement, reducing noise and correcting color distortions to produce cleaner inputs for SLAM pipelines.

### 7.5. Datasets for Underwater SLAM

DL models require substantial data for training, validation, and testing. Recent research has focused on providing comprehensive datasets covering various underwater scenarios while also providing ground-truth poses to train and evaluate DL models. For instance, Ferrera et al. [150] introduce a comprehensive dataset called Aqualoc to enhance SLAM methods for underwater vehicles operating near the seabed. This dataset, recorded in diverse environments, such as harbors and archaeological sites at depths of 270 m and 380 m, includes synchronized data from a monocular camera, an IMU, and a pressure sensor. It is available as robot operating system (ROS) bags and raw data, providing offline computed trajectories enabling benchmarking real-time localization methods. This dataset promotes significant advancements in underwater vision-based localization.

Another commonly used dataset for underwater SLAM evaluation is the EuRoC dataset [151]. Although originally captured in a terrestrial environment using a drone, the EuRoC dataset remains relevant for underwater research due to its inclusion of challenging conditions that sample those encountered underwater. It features sequences with variable lighting, motion blur, and diverse noise that simulate underwater challenges such as low visibility due to turbidity, dynamic lighting from surface reflections, and sensor noise from water particulates. These similarities allow researchers to test the robustness of SLAM algorithms under scenarios where direct underwater data may be limited. Furthermore, the scarcity of large-scale, publicly available underwater datasets with ground-truth poses enhances the EuRoC dataset’s utility as a proxy for benchmarking. While underwater-specific datasets like Aqualoc are ideal for direct relevance, the EuRoC dataset’s established benchmarks and challenging conditions make it a valuable and often necessary resource for assessing the performance and generalization of SLAM methods in underwater contexts.

Moreover, addressing the issue of underwater imaging affected by uneven lighting and scattered light, Shivaswamy et al. present a dataset of 1000 images with depth maps from a black smoker field at a depth of 1400 m. This study compares classical Markov random field-based segmentation and DL-based U-Net segmentation for detecting free space and enhancing clean mapping and navigation in complex underwater terrains [152]. Wang et al. [153] introduce a new dataset created with a controllable AUV equipped with high-precision sensors, including fiber-optic inertial sensors, DVL, and depth sensors. This rigorously tested dataset provides valuable data for evaluating navigation algorithms based on actual and calculated positions, focusing on the challenges of weak textures and image degradation in underwater environments.

## 8. Underwater SLAM Systems Evaluation and Comparison

Evaluating the performance of underwater SLAM systems is crucial to understanding their effectiveness in challenging underwater environments. Underwater SLAM evaluation typically involves two critical components: mapping evaluation and localization evaluation.

### 8.1. Mapping Evaluation

Mapping evaluation assesses the quality and accuracy of the maps generated by SLAM systems. Key considerations include map consistency, ensuring that the map accurately represents the environment without distortions or inconsistencies. Standard metrics for mapping evaluation are the structural similarity index (SSIM), which measures the similarity between the generated map and a ground truth map, and the intersection over union (IoU), which evaluates the overlap between the mapped areas and the actual environment [154].

To evaluate how accurately a SLAM system reconstructs the underwater environment, mapping metrics focus on comparing the generated map to a reference or ground truth representation.

**Structural Similarity Index:** The SSIM quantifies the similarity between two images (in this case, the generated map and the ground truth map). It considers luminance, contrast, and structural information to produce a value in the range [−1,1], where 1 indicates perfect similarity.

A simplified form of the SSIM between two images *X* and *Y* is given by

(1) $$\mathrm{SSIM}\left(X,Y\right)=\frac{\left(2\mu_{X}\mu_{Y}+C_{1}\right)\left(2\sigma_{XY}+C_{2}\right)}{\left(\mu_{X}^{2}+\mu_{Y}^{2}+C_{1}\right)\left(\sigma_{X}^{2}+\sigma_{Y}^{2}+C_{2}\right)}$$

where $\mu_{X}$ and $\mu_{Y}$ are the mean intensities of *X* and *Y*, $\sigma_{X}^{2}$ and $\sigma_{Y}^{2}$ are their variances, $\sigma_{XY}$ is the covariance, and $C_{1}$, $C_{2}$ are small constants to avoid division by zero.

**Intersection over Union:** The IoU measures the overlap between the mapped regions and the ground truth areas. It is defined as the ratio of the intersecting region to the union of the predicted and ground truth sets of mapped points:

(2) $$\mathrm{IoU}\left(A,B\right)=\frac{\left|A\cap B\right|}{\left|A\cup B\right|}$$

where *A* is the set of points mapped by the SLAM system, and *B* is the set of ground truth points. An IoU closer to 1 indicates a better overlap between the generated and actual maps.

These mapping metrics help determine if the SLAM-generated maps are free of distortions and inconsistencies, ensuring that the environment is accurately represented.

### 8.2. Localization Evaluation

Localization evaluation focuses on the accuracy of the robot’s estimated position and orientation within the environment.

#### 8.2.1. Traditional SLAM Metrics

Standard metrics used to evaluate SLAM performance include absolute pose error (APE) [155], which measures the difference between the estimated position and the ground truth; relative pose error (RPE), which evaluates the error relative to the actual position [156]; root mean squared error (RMSE) [155], which provides an aggregate measure of the errors over all *N* instances; and absolute trajectory error (ATE) [156]. All position vectors are measured in meters unless otherwise specified. Traditional SLAM metrics provide quantitative insights into system performance, enabling: accuracy assessment, error propagation analysis, and comparative evaluation.

**Absolute Pose Error:**

(3) $$APE=∥p_{\mathrm{pred}}-p_{\mathrm{true}}∥$$

where

- $p_{\mathrm{pred}}$ is the predicted position vector;
- $p_{\mathrm{true}}$ is the true position vector;
- $∥\cdot ∥$ denotes the Euclidean norm.

This metric provides direct insight into the accuracy of the SLAM system’s instantaneous pose estimates.

**Relative Pose Error:**

(4) $$RPE=\frac{∥p_{\mathrm{pred}}-p_{\mathrm{true}}∥}{∥p_{\mathrm{true}}∥}$$

This metric calculates the error relative to the magnitude of the actual position, providing a normalized measure of performance that is dimensionless.

**Root Mean Squared Error:**

(5) $$RMSE=\sqrt{\frac{1}{N}\sum_{i=1}^{N}{∥p_{\mathrm{pred}}^{\left(i\right)}-p_{\mathrm{true}}^{\left(i\right)}∥}^{2}}$$

where

- *N* is the total number of samples;
- $p_{\mathrm{pred}}^{\left(i\right)}$ and $p_{\mathrm{true}}^{\left(i\right)}$ are the predicted and true positions at instance *i*.

RMSE provides an aggregate measure of the errors over all instances, with larger errors having a more significant impact due to the squaring operation.

**Absolute Trajectory Error:**

(6) $$ATE_{RMSE}=\sqrt{\frac{1}{N}\sum_{i=1}^{N}{∥T_{\mathrm{pred}}^{\left(i\right)}-T_{\mathrm{true}}^{\left(i\right)}∥}^{2}}$$

where

- $T_{\mathrm{pred}}^{\left(i\right)}$ and $T_{\mathrm{true}}^{\left(i\right)}$ are the predicted and actual poses (including both position and orientation) at instance *i*.

ATE measures the difference between the estimated and ground truth trajectories over time, providing a global sense of the SLAM system’s accuracy.

**Sampling Rate:** The frequency at which sensor data are collected influences the resolution and responsiveness of the SLAM system. For example, a higher sampling rate (e.g., 30 Hz) can capture rapid movements more effectively but may increase the computational load.

**Trajectory Length:** The total distance or duration over which the SLAM system is evaluated. Longer trajectories can help assess the accumulation of errors over time, which is critical for understanding long-term navigation performance.

**Environmental Conditions:** Parameters such as water turbidity, lighting conditions, and dynamic obstacles affect sensor measurements and, consequently, the performance of SLAM. For example, higher turbidity levels can degrade visual sensor data, increasing positional errors.

**Sensor Specifications:** The characteristics of the sensors (e.g., camera resolution, IMU accuracy) impact the quality of the data and the SLAM system’s ability to estimate poses accurately. For instance, a high-resolution camera can provide more detailed visual information, improving feature detection and matching.

#### 8.2.2. DL-Based SLAM Metrics

In learning-based underwater SLAM systems, especially those involving DL, evaluation often goes beyond traditional pose errors. While standard localization metrics (APE, RPE, ATE, etc.) remain highly relevant, researchers have introduced or adapted additional metrics to better assess how well a DL-based SLAM system recovers motion and structure under challenging underwater conditions.

The odometry loss function, employed in DL-based SLAM applications, comprises two primary terms, each scaled by specific weighting factors to prioritize critical components. The first term is the positional loss ($L_{\mathrm{position}}$), expressed as

(7) $$L_{\mathrm{position}}=\frac{1}{N}\sum_{i=1}^{N}{\left|p_{\mathrm{pred}}^{\left(i\right)}-p_{\mathrm{true}}^{\left(i\right)}\right|}^{2}$$

This equation utilizes mean squared error to measure the difference between predicted ($p_{\mathrm{pred}}$) and accurate positional vectors ($p_{\mathrm{true}}$) in all *N* instances.

**Number of Instances (***N***):** This parameter indicates the number of data points over which the loss is computed. It depends on factors such as the sensor sampling rate and the mission duration. For instance, with a higher sampling rate or longer mission duration, *N* increases, providing a more extensive dataset for error calculation.

The second term is the angular error (Δψ), calculated as

(8) $$\Delta \psi =\psi_{\mathrm{pred}}-\psi_{\mathrm{true}}$$

and normalized within the interval [−π,π] using

(9) Δψ=(Δψ+π)mod2π−π

This ensures consistency in error measurement due to the periodic nature of angular data.

**Normalization of Angular Errors:** Normalizing Δψ within [−π,π] ensures that the angular differences are measured correctly, accounting for the circular nature of the rotational data. This prevents discontinuities in error measurements when the angles overlap, such as transitioning from $359^{\circ }$ to $0^{\circ }$.

The angular loss term ($L_{\mathrm{angle}}$) is defined as

(10) $$L_{\mathrm{angle}}=\frac{1}{N}\sum_{i=1}^{N}{\left(\Delta \psi^{\left(i\right)}\right)}^{2}$$

This represents the average squared angular errors across all instances.

The total odometry loss ($L_{\mathrm{odometry}}$) combines these terms:

(11) $$L_{\mathrm{odometry}}=w_{\mathrm{position}}L_{\mathrm{position}}+w_{\mathrm{angle}}L_{\mathrm{angle}}$$

where $w_{\mathrm{position}}$ and $w_{\mathrm{angle}}$ are weighting factors that balance the contributions of positional and angular errors.

**Weighting Factors ($w_{\mathrm{position}}$, $w_{\mathrm{angle}}$):** These parameters determine the relative importance of positional versus angular errors in the loss function. For example, if precise positioning is more critical than orientation in a particular application, a higher value may be assigned to $w_{\mathrm{position}}$ compared to $w_{\mathrm{angle}}$. Adjusting these weights allows for tailoring the SLAM system to prioritize certain aspects of performance.

**Beyond the basic odometry loss (positional and angular terms)** described earlier, state-of-the-art literature commonly employs segment-based evaluation metrics and scale-drift assessments, reflecting practices from the VO and SLAM communities [157,158]. These metrics help capture specific aspects of trajectory estimation quality that are highly relevant when learning-based approaches are applied in complex underwater scenarios.

**Segment-based Drift Metrics:** Inspired by terrestrial benchmarks (e.g., KITTI), segment-based metrics evaluate how the trajectory error accumulates over fixed distances or segments of the trajectory. For example, translational and rotational errors can be computed per 100 m or per fixed intervals. These evaluations produce metrics such as average translational drift (% per 100 m) and average rotational drift (°/100 m) [159]. These segment-based metrics highlight how well a DL-based SLAM system maintains consistency over longer traverses, a critical aspect in feature-poor underwater environments.

**Scale Drift:** Scale drift refers to the gradual scaling inconsistency that accumulates in monocular or learning-based SLAM systems. While some DL methods attempt to learn scale from stereo or depth data, purely monocular systems might suffer from drifting scales over time. Evaluating the percentage of scale error over extended trajectories or comparing learned scale estimates against ground truth depths can help quantify how effectively the DL-based SLAM maintains metric consistency [160].

**Feature-based Accuracy:** For some DL-based methods that learn feature extraction end-to-end, additional metrics can include feature repeatability and matching precision. Although more common in feature evaluation than full SLAM systems, these metrics can indirectly inform how the learned front-end influences overall SLAM accuracy [161]. Improved feature repeatability and robustness to underwater image degradation can lead to reduced trajectory error downstream.

DL-based SLAM metrics often combine odometry losses (positional and angular) to assess the quality of learned pose estimation directly, segment-based drift measures to understand long-term consistency and cumulative errors, scale drift assessments to ensure metric correctness, especially in monocular settings, or feature-based evaluations to examine the quality of learned visual representations.

In summary, evaluating underwater SLAM involves assessing both mapping and localization performance. Mapping metrics, such as SSIM and IoU, measure how accurately the SLAM-generated map reflects the true environment. Localization metrics such as APE, RPE, RMSE, and ATE gauge how closely the estimated trajectory matches the true trajectory. Additional factors like sensor sampling rate, environmental conditions, and sensor capabilities influence these metrics. Accurate localization is vital for underwater SLAM, as errors in position estimation can lead to significant deviations in the generated map. Therefore, this section focuses on localization evaluation and comparison.

### 8.3. Comparison of Underwater SLAM Methods

Underwater SLAM systems have undergone significant advancements, leading to a variety of traditional and deep learning-based approaches. These methods are evaluated against key metrics such as trajectory error and robustness across different datasets and environments. This section presents a comparative analysis of traditional and deep learning-based SLAM techniques, highlighting their performance, strengths, and limitations in underwater conditions.

While trajectory error and robustness assess accuracy, computational efficiency (FPS, memory usage) and power consumption determine the feasibility of SLAM methods in underwater deployments. FPS reflects real-time performance, critical for dynamic environments; memory usage impacts hardware selection on resource-constrained robots; and power consumption affects mission duration due to limited battery capacity. Unfortunately, many cited studies do not report these metrics, as indicated by "NR" (not reported) in Table 1 and Table 2. This gap highlights a need for standardized reporting in the field. Future research should include these metrics to enable comprehensive comparisons and optimize SLAM systems for underwater operations.

Traditional underwater SLAM methods often rely on classical filtering and optimization techniques adapted to work with data from sensors like sonar, cameras, and IMUs. Table 9 summarizes the performance of these methods across different datasets. However, the reliance on handcrafted features and the inability to adapt dynamically to challenging underwater environments limit these methods. For example, McConnell et al. (2022) [162] and Mu et al. (2022) [163] achieved relatively high RMSE values due to the noisy nature of acoustic and visual data, respectively, in underwater SLAM systems.

Deep learning-based approaches have emerged as a powerful alternative, leveraging neural networks to enhance feature extraction, pose estimation, and mapping. Table 10 provides a detailed comparison of these methods evaluated across diverse underwater datasets. Despite these advancements, the performance of deep learning methods varies significantly across datasets. This variation reflects the influence of environmental factors such as turbidity, lighting, and feature richness. Methods specifically designed for underwater conditions, such as RU-SLAM, outperform general-purpose SLAM techniques.

**Figure 15:**
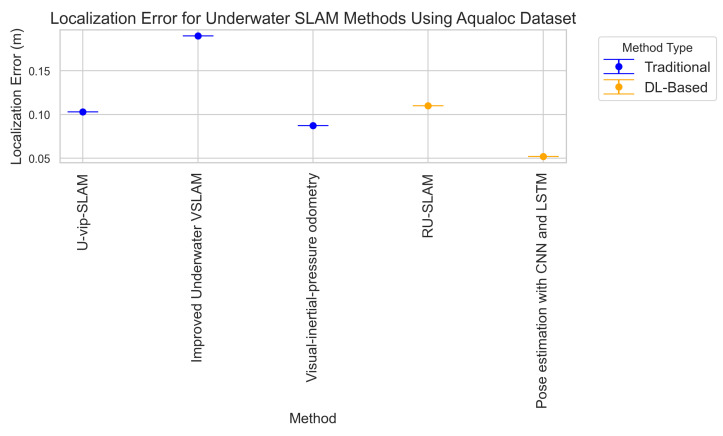
Localization error for underwater SLAM methods using the Aqualoc dataset. Traditional methods are shown in blue, and DL-based methods are shown in orange. Multi-sensor fusion approaches outperform vision-only methods, while DL-based methods generally achieve lower errors.

The localization error for various underwater SLAM methods using the Aqualoc dataset is shown in Figure 15. Traditional methods are highlighted in blue, while DL-based methods are highlighted in orange. This comparison provides an evaluation of their performance in the same dataset.

#### 8.3.1. Traditional Methods

Traditional SLAM methods demonstrate varying performance on the Aqualoc dataset. Multi-sensor fusion approaches, such as U-vip-SLAM and Visual-inertial-pressure odometry, achieve lower localization errors (0.103 m and 0.0873 m, respectively) compared to vision-only methods like *Improved Underwater VSLAM* (0.19 m). This highlights the importance of integrating multiple sensor modalities, for example visual, inertial, and pressure data, to improve localization accuracy in underwater environments.

#### 8.3.2. DL-Based Methods

DL-based methods, such as *RU-SLAM* and *pose estimation with CNN and LSTM*, generally outperform traditional methods on the Aqualoc dataset. For example, *pose estimation with CNN and LSTM* achieves the lowest localization error (0.0519 m), demonstrating the effectiveness of deep learning techniques in feature extraction and pose estimation. However, *RU-SLAM* shows a slightly higher error (0.110 m) compared to some traditional methods, indicating that the performance of DL-based methods can vary depending on the specific architecture and training approach.

#### 8.3.3. Analysis on Why DL Outperforms

DL’s superiority stems from three key capabilities:

- Feature Learning: DL automatically learns robust features, such as Wang 2024’s CNN-attention hybrid, which adapts to turbidity variations, unlike handcrafted features in traditional methods that fail in low visibility.
- Non-linear Modeling: Recurrent architectures (LSTM/GRU) in DL methods like Sudevan 2023 better model complex underwater dynamics.
- Multi-modal Fusion: DL’s learned fusion, such as Jang 2021’s opti-acoustic network, outperforms traditional sensor weighting by discovering complementary sensor relationships.

However, traditional methods remain preferable when training data are scarce, computational resources are limited, or environments match the method’s assumptions, for example, structured pipelines where geometry-based SLAM suffices.

## 9. Research Gaps and Opportunities

Despite significant advancements in underwater SLAM, a number of critical challenges remain that limit widespread adoption and reliability. These challenges stem from the unique conditions in underwater environments—ranging from limited visibility and sensor noise to bandwidth-constrained communication channels. Addressing these gaps is vital not only to enhance the robustness and accuracy of SLAM algorithms, but also to enable a broader suite of applications, including long-term monitoring, resource management, and large-scale cooperative missions. In the following subsections, we examine the current limitations in underwater SLAM research, discuss emerging sensor and computing technologies, and highlight how novel machine learning paradigms such as deep reinforcement learning and large language models can push the boundaries of performance and functionality in this critical domain.

### 9.1. Gaps in Underwater SLAM Research

#### 9.1.1. Limitations of Existing Underwater SLAM Datasets

While datasets such as Aqualoc [150] and the black smoker field dataset [152] have provided valuable resources for training and validating SLAM algorithms, critical challenges still remain:

- **Limited Diversity:** Current datasets often cover a narrow range of underwater conditions and depth profiles. Factors such as turbidity, lighting variations, and diverse seafloor terrains are not always comprehensively represented, limiting the robustness of deep learning-based SLAM methods when deployed in new environments.
- **Insufficient Annotations:** Many underwater datasets lack detailed ground-truth information, particularly for semantic segmentation or dynamic object tracking. This hampers the development of advanced DL techniques that rely on precise, fine-grained annotations.
- **Specialized Use Cases:** Some datasets focus on specific tasks such as archaeological surveys or sensor configurations like monocular cameras only, reducing their general applicability. This specialization can make it difficult to benchmark SLAM algorithms intended for multi-sensor or cooperative AUV scenarios.

To advance the field, there is a pressing need for more extensive, standardized datasets that capture the full spectrum of underwater environments that could help train robust SLAM models capable of generalizing to unseen conditions. Furthermore, improved annotations such as semantic labels and precise depth maps would enable the application of cutting-edge deep learning techniques that go beyond basic pose estimation and mapping to semantic and dynamic scene understanding.

#### 9.1.2. Absence of Evaluation Metrics Tailored for Underwater Environments

Standard SLAM metrics, such as ATE or RPE, are widely used in terrestrial settings but do not always capture the unique challenges faced underwater. For instance, low visibility, sensor drift, and fluctuating lighting conditions can lead to highly non-Gaussian noise distributions, which are not adequately characterized by traditional metrics alone. Developing novel evaluation protocols and error metrics capable of assessing robustness under these distinct conditions is critical to advancing underwater SLAM. Incorporating environment-specific factors, such as turbidity levels or salinity-induced sensor bias, into metric design could yield more meaningful performance assessments.

#### 9.1.3. Resource-Constrained Real-Time Computation

Underwater robots often operate with limited computational power and battery capacity, making it challenging to run resource-intensive SLAM algorithms in real-time. Deep learning-based methods typically demand high GPU processing, while energy availability is restricted for extended underwater missions. Consequently, approaches that excel in laboratory conditions may fail to meet real-time or energy requirements in the field. Methods such as neural network pruning, quantization, and model compression, along with optimized sensor scheduling, must be developed and standardized to enable practical, long-duration deployments. Further research is required to balance accuracy with computational feasibility, ensuring that underwater SLAM solutions can be effectively implemented on low-power, embedded systems.

#### 9.1.4. Cost and Power Consumption of Sensors

Underwater SLAM faces significant challenges due to the complex underwater environment, characterized by limited visibility, variable conditions, and restricted GNSS availability. This puts high demands on exteroceptive sensors (sonars, cameras, and DVLs) as well as on proprioceptive sensors (IMU), to enhance the navigational accuracy and resilience of unmanned underwater vehicles. This becomes a major concern, particularly for long-duration missions.

A study by Merveille et al. analyzes sensor fusion techniques, combining proprioceptive and exteroceptive sensors to enhance UUV navigation [170]. They explore the use of sensors, including IMUs, DVLs, cameras, sonar, and LiDAR, and further enhance their capabilities through various filtering methods. The results are compared to computationally efficient emerging technologies such as quantum sensors and AI-driven filtering. The power consumption for such systems presents itself as a bottleneck for wider applications. The study highlights the trade-offs between accuracy, computational and power demands, and adaptability according to environmental changes.

Another related limitation in the use of underwater sensors for navigation and SLAM is the high cost of sensors operating in difficult environments. Acoustic modems and positioning systems have been designed for military and oil and gas industries, requiring deep water deployments and high reliability, making them expensive and unsuitable for low-cost applications. However, recent advancements in low-cost unmanned vehicles like ROVs and AUVs for shallow water missions and the need for sensor networks to monitor water quality and climate change effects have driven the development of low-cost, low-power acoustic modems and positioning systems.

In a study on navigation techniques for inspection and data acquisition in UWSNs, Wibisono et al. discussed implementing a dynamic homing control algorithm in AUVs, which helps enhance data acquisition by directing movement based on the importance of information at each point. This approach not only improves observation accuracy but also optimizes time and power usage, ensuring the collected data are of maximum value for mission or research objectives with the least power required for sensors and AUV navigation.

A study by Campagnaro et al. found that low-cost sensors with reduced transmission and positioning range and precision are actually suitable for shallow water environments where obstacles limit long-range transmissions [171]. The paper further reviews recent developments in low-cost acoustic communication and positioning systems, analyzing university prototypes and commercial devices, and exploring potential new applications. The study concludes by urging developers to focus on the growing demand for low-cost sensors, especially for swarm applications, and calls on the research community to provide easy-to-understand and implement manuals for these sensors.

SVIn2 is a novel SLAM system designed by Rahman et al. for challenging underwater environments, focusing on cost savings by reducing the number of necessary sensors [74]. Unlike previous systems that require DVL or expensive INS, SVIn2 uses a unique sensor configuration where the mechanical scanning sonar maps the vertical plane parallel to the image plane, enabling the mapping of cave structures. It integrates scanning profiling sonar, visual, inertial, and water-pressure data in a non-linear optimization framework. The open-source software has been validated in benchmark datasets and real-world scenarios, showing excellent accuracy and robustness.

#### 9.1.5. Limited Communication for Cooperative SLAM

Despite the promise of multi-robot SLAM in large-scale or complex underwater missions, effective collaboration remains constrained by the low bandwidth and high latency of underwater communication channels, predominantly acoustic. Transmitting raw sensor data or high-frequency map updates across multiple AUVs is often infeasible, leading to incomplete or inconsistent shared maps. Innovative communication strategies—such as exchanging compressed feature representations or selectively transmitting critical map segments—are needed to overcome these limitations. Developing robust protocols for multi-agent coordination, fault-tolerance, and bandwidth adaptation will be essential for enabling efficient, collaborative SLAM in real-world marine applications.

### 9.2. Opportunities for Future Research

Underwater SLAM is a rapidly evolving field with huge potential for innovation. While progress has been made, several emerging applications and research directions offer exciting opportunities to push the boundaries of what is possible. This section outlines key areas where future research can drive advancements in underwater SLAM, addressing both current limitations and new frontiers.

#### 9.2.1. Advancing Deep Reinforcement Learning for Underwater SLAM

Deep reinforcement learning has shown great promise in enhancing active localization and visual navigation in robotics. However, its application in underwater SLAM remains challenging due to the unique conditions of underwater environments, such as light absorption, scattering, and fluid dynamics [172]. Future research should focus on the following aspects:

- **Realistic Simulation Environments:** developing high-fidelity underwater simulation environments that accurately model underwater physics, including light absorption, scattering, and fluid dynamics, to train DRL models effectively;
- **Sample Efficiency:** improving sample efficiency through transfer learning, meta-learning, and sim-to-real approaches to reduce the reliance on large, costly datasets;
- **Hybrid DRL Frameworks:** integrating model-based and model-free reinforcement learning techniques to enhance data efficiency and adaptability;
- **Hierarchical DRL:** leveraging hierarchical DRL approaches to decompose complex tasks into smaller, manageable sub-tasks, improving scalability and robustness;
- **Adaptability to Dynamic Conditions:** ensuring DRL models can adapt to varying underwater conditions, such as changes in turbidity, dynamic obstacles, and environmental disturbances, through robust policy learning and domain adaptation methods.

#### 9.2.2. Advancing Transformer-Based Underwater SLAM

Transformer architectures have revolutionized computer vision and robotic perception, offering superior performance in modeling long-range dependencies compared to traditional CNN-based approaches. Their application to underwater SLAM presents significant opportunities to address key challenges in feature association and dynamic environment handling [173]. Future research should focus on the following aspects:

- **Efficient Transformer Designs:** developing lightweight transformer variants optimized for resource-constrained underwater vehicles;
- **Multi-Modal Fusion:** exploring cross-attention mechanisms to effectively combine visual, acoustic, and inertial sensor data, improving robustness in turbid conditions where single modalities fail [174];
- **Self-Supervised Learning:** developing transformer-based self-supervised approaches that can learn robust feature representations from limited labeled underwater data [175].

#### 9.2.3. Enhancing 3D Reconstruction and Semantic Mapping

Semantic SLAM, which combines traditional geometric mapping with semantic understanding, offers richer representations of the environment by identifying and categorizing elements within the map [176,177]. Future research should focus on the following aspects:

- **Optical Distortion Compensation:** developing algorithms that compensate for underwater optical distortions, enabling more accurate 3D reconstructions from monocular or stereo-vision systems;
- **Underwater Semantic Segmentation:** creating specialized underwater semantic segmentation datasets to train robust object recognition and semantic segmentation models tailored for underwater conditions;
- **Graph Neural Networks (GNNs):** exploring GNNs to capture spatial relationships between objects within underwater environments, providing a richer semantic understanding;
- **Real-Time Performance:** optimizing algorithms for efficiency and leveraging specialized hardware accelerators to achieve real-time performance in 3D reconstruction and semantic mapping on resource-constrained underwater vehicles.

#### 9.2.4. Integrating Large Language Models for Underwater SLAM

LLMs have emerged as powerful tools for semantic understanding and natural language interactions. In the broader SLAM domain, LLMs have already been employed to enhance visual place recognition and localization. For example, LP-SLAM [178] leverages LLMs to detect text in scenes and use it as landmarks for mapping and localization. Similarly, FM-Loc [179] integrates foundation models, including LLMs, to improve place recognition by incorporating semantic reasoning into the SLAM pipeline, enhancing robustness in complex environments.

In underwater contexts, LLMs have demonstrated significant promise. MarineInst [180] employs vision-language models to achieve semantic instance understanding, enabling underwater robots to interpret their surroundings at a semantic level, such as identifying objects or features in the environment. ChatSim [181] integrates LLMs with underwater simulations, providing intuitive natural language control of simulated environments, which could be extended to real-world underwater systems.

These examples collectively illustrate how LLMs can improve underwater SLAM by enabling semantic understanding, supporting natural language interfaces, and facilitating data integration. Future research should focus on the following aspects:

- **Efficient Deployment Strategies:** developing compact representations of LLMs through model distillation and quantization to enable their deployment on resource-constrained underwater SLAM systems;
- **Multimodal LLMs:** advancing multimodal LLMs that integrate acoustic, visual, and textual data for a holistic understanding of underwater environments;
- **Natural Language Interfaces:** exploring the use of LLMs to enable natural language control and interaction with underwater SLAM systems, improving usability and accessibility.

#### 9.2.5. Addressing Dynamic Environments

Dynamic underwater environments, with moving objects such as marine life or floating debris, pose significant challenges for SLAM systems. Future research should focus on the following aspects:

- **Dynamic Object Filtering:** developing deep learning-based object tracking methods, such as attention-based transformers, to detect and filter dynamic objects from SLAM computations, preventing map corruption and maintaining accuracy [168,182];
- **Environmental Awareness:** enhancing SLAM systems with environmental awareness capabilities to adapt to real-time changes in conditions such as water clarity and light levels;
- **Predictive Models:** integrating predictive models of underwater environments that can anticipate and compensate for dynamic changes, improving overall robustness.

#### 9.2.6. Overcoming Communication Limitations in Cooperative SLAM

Collaborative SLAM systems are transforming multi-robot operations, enhancing scalability and flexibility in underwater exploration. However, communication constraints pose a significant challenge [183]. Systems such as Swarm-SLAM enable multiple robots to work together, sharing mapping information to improve the overall environmental understanding. Future research should focus on the following aspects:

- **Alternative Communication Technologies:** exploring optical or electromagnetic communication methods to complement traditional acoustic channels, improving bandwidth and reliability;
- **Machine Learning-Based Predictive Communication:** designing predictive communication models to optimize data exchange strategies and adjust bandwidth allocation dynamically, enhancing the resilience of cooperative SLAM systems;
- **Distributed SLAM Frameworks:** developing robust distributed SLAM frameworks that enable efficient information exchange and collaborative mapping among multiple robots [162,184].

#### 9.2.7. Exploring New Sensor Technologies

The development of novel sensor technologies has the potential to significantly enhance underwater SLAM capabilities. Future research should focus on the following aspects:

- **Event Cameras:** developing SLAM systems using neuromorphic event cameras that asynchronously detect brightness changes at microsecond resolution, enabling high dynamic range perception in low-light conditions with minimal power consumption [185];
- **Bio-Inspired and Quantum Sensors:** investigating bio-inspired [186] or quantum [187] sensors that can operate effectively underwater, offering new avenues for SLAM enhancement;
- **Sensor Miniaturization:** reducing the size and energy consumption of sensors to make them more suitable for small, battery-powered underwater vehicles [188];
- **Hyperspectral Imaging:** advancing hyperspectral imaging sensors to provide detailed spectral information, enhancing feature detection and scene segmentation in underwater SLAM;
- **Energy Harvesting:** incorporating energy-harvesting technologies, such as piezoelectric materials, to power onboard sensors through underwater vibrations or currents, extending operational duration;
- **Environmental Sensors:** integrating environmental sensors that monitor parameters such as salinity, temperature, and pH, provides valuable contextual data to improve the accuracy and reliability of SLAM systems.

Continued research into these areas will be critical for overcoming the current limitations of underwater SLAM, enhancing collaboration among autonomous systems, integrating advanced technologies that enable more efficient and effective underwater exploration and navigation, and, so, revolutionizing several emerging applications, including underwater archaeology, marine biology and ecology, offshore infrastructure inspection, disaster response and recovery, and deep-sea mining.

## 10. Conclusions

This survey has provided a comprehensive examination of the integration of deep learning (DL) into simultaneous localization and mapping (SLAM) for underwater applications. As underwater navigation remains a challenge due to limited visibility, sensor noise, and the unpredictable nature of marine environments, traditional SLAM techniques have struggled to achieve robust and reliable performance. By leveraging DL, researchers have made significant strides in improving feature extraction, image enhancement, and sensor fusion, enabling more precise localization and mapping in underwater settings. Through a critical analysis of existing methodologies, this review has highlighted key advancements in DL-based underwater SLAM, including its role in enhancing front-end perception, mitigating environmental distortions, and improving loop closure detection. The discussion has also underscored the importance of integrating multi-modal sensor data, such as optical, sonar, acoustic, and inertial measurements, to compensate for the inherent limitations of individual sensors. Furthermore, this survey introduced a novel classification framework for underwater SLAM based on the integration of underwater wireless sensor networks (UWSNs), emphasizing the transformative potential of communication-aware SLAM systems. By leveraging distributed sensing and acoustic communication, UWSNs facilitate collaborative mapping and real-time localization among multiple autonomous underwater vehicles, leading to more scalable and resilient SLAM solutions. Despite these advancements, several challenges remain that require further exploration. The development of adaptive learning models capable of dynamically adjusting to variations in lighting, turbidity, and marine conditions is essential for ensuring consistent performance across different environments. Moreover, achieving real-time deployment of DL-based SLAM on computationally constrained platforms remains an open problem, necessitating the optimization of deep neural architectures for efficiency and energy conservation. Another pressing challenge is the need for long-term SLAM solutions that can maintain robustness over extended missions, addressing drift correction, large-scale mapping, and global consistency. Addressing these issues will be pivotal in driving the next generation of autonomous underwater robots capable of executing complex tasks with minimal human intervention. By synthesizing the latest research and identifying emerging trends, this survey serves as a valuable resource for researchers and practitioners working at the intersection of SLAM, deep learning, and underwater robotics. The continued evolution of DL-powered SLAM frameworks will play a crucial role in advancing underwater exploration, enabling more reliable navigation, resource mapping, and environmental monitoring. As the field progresses, further interdisciplinary collaboration will be key to overcoming existing barriers and unlocking new possibilities for autonomous operations in the vast and uncharted underwater world.

## Figures and Tables

**Table 1:** Comparison of Back-end SLAM Approaches.

Aspect	Filtering-Based	Optimization-Based
Core Principle	Probabilistic state estimation	Graph-based minimization
Key Methods	EKF/UKF SLAM Particle filter (FastSLAM) RBPF SLAM	Graph-based SLAM Factor-graph SLAM Bundle adjustment
Strengths	Online processing Computationally efficient Handles real-time updates	Higher accuracy Better loop closure Handles non-linearities
Limitations	Linearization errors Particle degeneracy Drift accumulation	Higher computation Batch processing needed Memory intensive

**Table 3:** Comparison of sensors for underwater SLAM.

Sensor Type	Advantages	Disadvantages	Use Cases
Vision	High-resolution imagery, rich texture/color information, suitable for object recognition	High computational load, limited recognition accuracy in turbid water, short effective range, sensitive to lighting conditions	Archaeological site documentation, coral reef monitoring, pipeline inspection, visual odometry in clear waters
LiDAR	Millimeter-level depth accuracy, works in complete darkness, unaffected by turbidity	High power consumption, limited by absorption, sensitive to scattering particles	High-precision 3D mapping, ship hull inspection, underwater structure measurement
Sonar	Long range, works in zero visibility and in the presence of particles	Low angular resolution, multipath interference, slow refresh rate	Deep-sea exploration, mine detection, large-area seabed mapping, navigation in featureless terrain

**Table 4:** Selected vision-based underwater SLAM systems.

Reference	Problem	Method	Findings	Limitations
Jung et al. (2017) [52]	SLAM for AUVs	Vision-SLAM with landmarks, IMU, DVL	Enhanced visual sensing	Requires specific sensors
Joshi et al. (2023) [56]	Robust estimation in low visibility	SM/VIO estimator switching	Tracked AUVs in featureless env.	Needs lighting adaptation
Lu et al. (2024) [53]	Navigation precision	ORB-SLAM3-VIP fusion	42% error reduction	Needs extra initialization
Ou et al. (2024) [51]	Passive V-SLAM improvement	Hybrid-VINS	Outperformed passive setups	Slow startup
Liu et al. (2024) [54]	Sediment visibility loss	Adaptive filtering V-SLAM	Better than ORB-SLAM3	Inflexible parameters

**Table 5:** Summary of selected underwater SLAM studies using acoustic sensors.

Reference	Challenge	Approach	Results	Limitations
He et al. (2012) [59]	AUV navigation with sonar	FastSLAM with mechanical scanning sonar	Improved mapping and localization	Relies on particle filters; limited generalization
Fallon et al. (2013) [61]	Feature reacquisition in shallow water	Feature-based navigation with forward-looking sonar	Effective, low-cost reacquisition	Limited to shallow water; prone to false positives
Machado et al. (2016) [62]	Loop closure detection	Gaussian probability models for topology/shape	Improved image matching	Complex; slow for real-time use
Valdenegro-Toro et al. (2017) [60]	Sonar image matching	CNN-based sonar image matching	High accuracy over traditional methods	Requires large datasets; computationally demanding
Choi et al. (2020) [67]	ASV navigation with sonar	Acoustic and terrain-based methods	Effective waypoint tracking and obstacle avoidance	Challenges in dynamic environments
Cheng et al. (2022) [63]	Real-time SLAM with sonar data	Filter-based SLAM with multi-beam sonar (RBPF)	Enhanced state estimation and mapping	Computational complexity; data handling issues
Nakamura et al. (2023) [64]	3D mapping with acoustic cameras	YOLOv7 and ICP-based SLAM	Improved 3D reconstruction and map accuracy	Limited by camera quality and underwater conditions
Hansen et al. (2024) [65]	Sonar data processing	MSS datasets with EKF	Provided baseline solutions for sonar SLAM	No dynamic scene ground truth; limited generalization
Yuan et al. (2017) [68]	Low-cost SLAM accuracy and stability	AEKF-SLAM with an augmentation phase	Reduced error accumulation and improved efficiency	Requires local-to-global map conversion

**Table 6:** Summary of selected sensor fusion research in underwater SLAM.

Reference	Challenge	Approach	Results	Limitations
Font et al. (2017) [72]	AUV navigation accuracy	EKF with USBL, IMUs, DVLs, and GNSS	Enhanced robustness	Requires precise calibration
Rahman et al. (2018) [73]	Underwater mapping	Visual-inertial SLAM with acoustic data	Improved 3D reconstruction	Limited sensor coverage
Thoms et al. (2021) [78]	Infrastructure inspection	LiDAR and sonar fusion	High-quality 3D maps	Complex sensor integration
Hu et al. (2022) [79]	Localization drift	Visual-inertial-pressure fusion	Reduced drift	Relies on pressure data
Cardaillac et al. (2023) [80]	Acoustic-optical matching	Camera and sonar fusion	Improved scale recovery	Requires calibration
Qiu et al. (2024) [81]	Illumination issues	Acousto-optic feature association	Improved trajectory accuracy	Depends on beam positions
Jang et al. (2021) [82]	Sensor mismatches	Opti-acoustic SLAM with style-transfer	Improved feature matching	Needs better natural object handling
Xu et al. (2021) [83]	Poor visibility	Visual-acoustic bundle adjustment	Enhanced robustness	Needs high-quality data
S. Ma et al. (2024) [75]	Scale estimation	Monocular-inertial-pressure fusion	Improved scale and pose accuracy	Requires stable pressure measurements
Y. Huang et al. (2024) [76]	Stability under variable conditions	Multi-sensor fusion with refined data association	Improved stability	Complex calibration

**Table 7:** Underwater communication spectrum for SLAM applications.

Method	Frequency/Range	Bandwidth	SLAM Use Case	Limitations
Acoustic	1–500 kHz	<100 kbps	Long-range mapping, DVL integration	Latency, multipath
Optical	450–700 nm	Up to Gbps	Clear-water VSLAM	Turbidity, line of sight
RF	<30 Hz	∼1 bps	Emergency signaling	low bandwidth
MI	10–100 kHz	<100 kbps	Short-range AUV teams	Limited range

**Table 8:** Summary of selected DL-based underwater SLAM: problem, method, DL architecture, findings, and limitations.

Reference	Problem	Method	DL Architecture	Findings	Limitations
Burguera et al. (2020) [115]	VSLAM	Unsupervised neural network	Autoencoder	Improved feature matching and pose estimation	May not scale well with large datasets
Torroba et al. (2020) [116]	SLAM point cloud registration	PointNet-based covariance estimation	PointNet	Enhanced point cloud registration accuracy	May require large datasets
Leonardi et al. (2020) [117]	VSLAM	CNN-based keypoint rejection system	CNN	Increased robustness in VSLAM	May not handle all types of keypoint failures
Marques et al. (2019) [118]	VSLAM	GAN-based depth estimation	GAN	Achieves trajectory error of 1.6 feet in subsea test dataset	Requires a dataset of videos
Burguera et al. (2022) [119]	Graph VSLAM	Outlier-resilient visual graph-SLAM	Siamese CNN	Reduces false loop closures	Has not been tested on AUV in real environment
Li et al. (2018) [120]	Real-time SLAM	Sonar imaging-based saliency-aware loop closure	CNN	Reliable data association	Specific to certain environments
Wang et al. (2022) [121]	Visual loop closure detection	Variational autoencoder network	VAE	Achieved recall rate of 92.31% in underwater dataset	Has not been tested on AUV in real environment
Tan et al. (2023) [122]	SLAM	Data-driven loop closure detection	Siamese CNN	Provides loop closure method and bathymetric dataset	Has not been tested in real environment

**Table 9:** Comparison of traditional methods in underwater SLAM.

Source	Method	Dataset	Metric (Meters)
Rahman et al. (2022) [74]	Multi-sensor fusion-based underwater SLAM	EuRoC	RMSE = 0.13
		cavern1	RMSE = 0.1243
		cavern2	RMSE = 0.1722
Zhang et al. (2022) [2]	CLAHE, median filtering (MF), and DCP image enhancement	Turbid underwater images	RMSE = 0.196
Amarasinghe et al. (2020) [164]	Monocular visual SLAM algorithm	Simulated Dataset and Real-world images	RMSE = 1.10
Zhang et al. (2024) [45]	Multisensor fusion integrating stereo vision, multibeam imaging sonar, and IMU	Images from water tank at Shanghai Jiao Tong University	RMSE = 0.018
Vargas et al. (2021) [165]	Visual SLAM fusing acoustic sensing	Custom dataset recorded in pool with and without lights	RMSE = 0.14
Billings et al. (2022) [166]	Fusing features from a camera into the map	UVMS collected images in Costa Rica	RMSE = 0.014
McConnell et al. (2022) [162]	Distributed robust acoustic communication-efficient SLAM for imaging sonar	Dataset at two sites in New York	RMSE = 1.29
Roznere et al.(2021) [167]	Monocular image depth estimation using single-beam echosounder	Custom recorded dataset with camera and echosounder	RSME = 0.169
Hu et al. (2022) [79]	Visual-inertial-pressure odometry	EuRoC	RMSE = 0.274
		Aqualoc	RMSE = 0.0873
Amarasinghe et al. (2024) [168]	U-vip-SLAM: underwater visual-	Aqualoc	RMSE = 0.103
	inertial-pressure SLAM	EuRoC	RMSE = 0.088
Leonardi et al. (2023) [169]	UVS: improved underwater VSLAM	Aqualoc	RMSE = 0.19
		RTMVO 04	RMSE = 1.10
Demim et al. (2022) [39]	Adaptive smooth variable structure filter (SVSF-SLAM) strategy	Experimental sea trials	RMSE = 0.9824
Mu et al. (2022) [163]	Variational Bayesian-AUFastSLAM	Experimental sea trials	RMSE = 1.753

**Table 10:** Comparison of deep learning-based underwater SLAM methods.

Reference	Method	Dataset	Metric
Teixeira et al. (2020) [136]	SLAM with CNN and LSTM	CRAS Pool	ATE = 0.071 m
		Urgeirica Mine	ATE = 0.111 m
Jang et al. (2021) [82]	Pose-graph SLAM with CNNs for opti-acoustic processing	Water tank tests	RMSE = 0.2917 m
McConnel et al. (2022) [147]	Sonar-based SLAM with CNN encoder–decoder architecture	Simulated underwater environment	RMSE = 0.95 m
Zheng et al. (2023) [129]	SLAM with GAN-enhanced	URPC	ATE = 1.344m, RMSE = 1.447 m
	underwater images	OUC Fisheye	ATE = 2.410 m, RMSE = 2.450 m
Xin et al. (2023) [127]	ULL-SLAM: SLAM with CNN-based underwater low-light enhancement	URPC-dark	ATE = 1.292m, RMSE = 1.316 m
Wang et al. (2024) [128]	RU-SLAM: CNN and	Aqualoc	APE = 0.110 m, RPE = 0.090 m
	attention for local and global descriptors	EuRoC	APE = 0.031 m
Lin et al. (2021) [135]	Pose estimation based on GRU	3 Sets of simulations (Averaged)	RMSE = 0.581 m
Sudevan et al. (2023) [137]	Pose estimation with 2D CNN, 1D CNN and LSTM	Aqualoc	RMSE = 0.0519
Li et al. (2024) [138]	Pose estimation with CNN with attention for IMU	Open sea trials sequences (Averaged)	RMSE = 9.707 m
Wang et al. (2022) [121]	Loop closure (LC) with	Indoor fire pool	LC average precision = 0.9945
	variational autoencoders	Yellow Sea trials	LC average precision = 0.9818
Tan et al. (2023) [122]	Loop closure (LC) with a Siamese network	Bathymetric point clouds	LC accuracy = 0.61
Abdullah et al. (2024) [149]	CaveSeg: semantic mapping with Swin Transformers	Underwater cave tests	IoU = 48.11%
